# Identifying Inhibitor-SARS-CoV2-3CL^pro^ Binding Mechanism Through Molecular Docking, GaMD Simulations, Correlation Network Analysis and MM-GBSA Calculations

**DOI:** 10.3390/molecules30040805

**Published:** 2025-02-10

**Authors:** Jianzhong Chen, Jian Wang, Wanchun Yang, Lu Zhao, Xiaoyan Xu

**Affiliations:** School of Science, Shandong Jiaotong University, Jinan 250357, China; wangjian_lxy@sdjtu.edu.cn (J.W.); yangwch1982@126.com (W.Y.); zhaolusdu@163.com (L.Z.); xuxiaoyan@sdjt.edu.cn (X.X.)

**Keywords:** SARS-CoV2-3CL^pro^, GaMD simulations, correlation network analysis, normal mode analysis, MM-GBSA

## Abstract

The main protease of the severe acute respiratory syndrome coronavirus 2 (SARS-CoV-2), known as 3CL^pro^, is crucial in the virus’s life cycle and plays a pivotal role in COVID-19. Understanding how small molecules inhibit 3CL^pro^’s activity is vital for developing anti-COVID-19 therapeutics. To this end, we employed Gaussian accelerated molecular dynamics (GaMD) simulations to enhance the sampling of 3CL^pro^ conformations and conducted correlation network analysis (CNA) to explore the interactions between different structural domains. Our findings indicate that a CNA-identified node in domain II of 3CL^pro^ acts as a conduit, transferring conformational changes from the catalytic regions in domains I and II, triggered by the binding of inhibitors (7YY, 7XB, and Y6G), to domain III, thereby modulating 3CL^pro^’s activity. Normal mode analysis (NMA) and principal component analysis (PCA) revealed that inhibitor binding affects the structural flexibility and collective movements of the catalytic sites and domain III, influencing 3CL^pro^’s function. The binding free energies, predicted by both MM-GBSA and QM/MM-GBSA methods, showed a high correlation with experimental data, validating the reliability of our analyses. Furthermore, residues L27, H41, C44, S46, M49, N142, G143, S144, C145, H163, H164, M165, and E166, identified through residue-based free energy decomposition, present promising targets for the design of anti-COVID-19 drugs and could facilitate the development of clinically effective 3CL^pro^ inhibitors.

## 1. Introduction

The global coronavirus disease pandemic in 2019, (COVID-19), triggered by severe acute respiratory syndrome coronavirus 2 (SARS-CoV-2), brought heavy threats on human health, and deaths [1,2,3,4], which greatly disturbs global economic development, as well as scientific and cultural exchange. Although some clinical treatments have been realized, their clinical efficacy is low and the corresponding sequela is identified, thus making continued research for additional therapeutics essential [5,6]. The recent studies indicated that the chymotrypsin-like protease (3CL^pro^), or main protease (M^pro^), and the papain-like protease (PL^pro^) play key roles in viral replication of SARS-CoV-2 [3,7,8,9,10]. The 3CL^pro^ or M^pro^ is a cysteine protease responsible for cleaving 11 distinct sites of the polyproteins into mature functional proteins [11] while the PL^pro^ is responsible for cleavage at three other unique sites [12]. The inhibition of the activity of 3CL^pro^ or M^pro^ and PL^pro^ prevents the formation of replication-essential enzymes and viral replication [13]. Among the two proteases, the 3CL^pro^ and spike protein are encoded in different regions of the viral genome, thus the 3CL^pro^ has been an attractive target for small-molecule oral therapeutics toward treatment of COVID-19.

**Figure 1:**
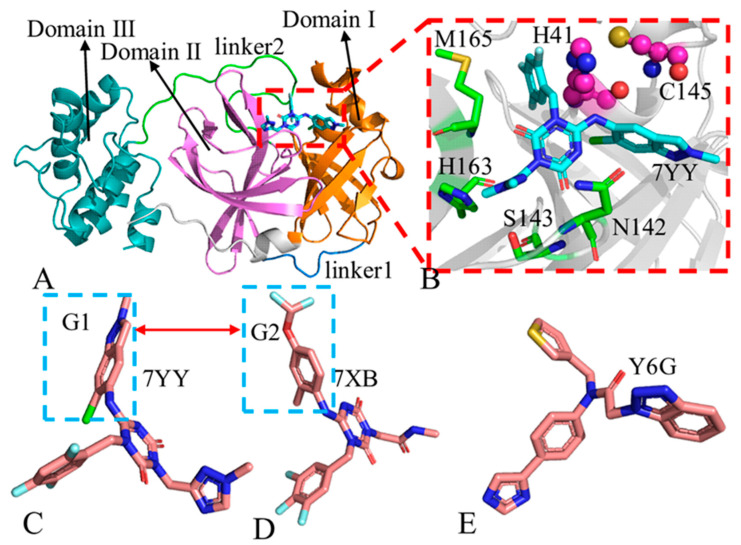
Molecular structures: (**A**) 7YY-3CLpro complex with domains I, II and III, in which 3CL^pro^ and 7YY are displayed in cartoon and stick modes, respectively, (**B**) key sites, in which a catalytic dyad is shown in ball and stick modes while significant residues are indicated in stick modes and (**C**), (**D**) and (**E**) correspond to structures of three inhibitors 7YY, 7XB and Y6G, respectively.

The 3CL^pro^ primarily exists in a dimer mode and belongs to the catalytically active species. In structural topology, COVID-19 3CL^pro^ consists of three structural domains (I–III) connected by flexible loops (Figure 1A), in which the structure (7VU6) in protein data bank (PDB) is used to depict the topology structure of 3CL^pro^ using the program PyMOL (www.pymol.org). Domains I and II are β-barrel domains that surround the catalytic active site region (residues C145 and H41), shown in Figure 1B. Domain III is a rich α-helical domain involved in the formation of dimerization. The catalytic residue C145 acts as a nucleophile and H41 acts as a base, able to activate the nucleophile by deprotonating the cysteine thiol [14]. The binding of inhibitors in the catalytic active sites can efficiently hold back the activity of 3CL^pro^. Previous studies have verified that peptide-like 3CL^pro^ inhibitors with reactive “warheads” have potent antiviral activities in vitro, and several drugs can reduce viral loads in vivo in SARS-CoV-2-infected human ACE2 transgenic mouse models [15,16], showing prospects of small molecule inhibitors in the treatment of COVID-19 [17,18].

To date, many researchers have focused on the development of inhibitors against COV 3CL^pro^ [19,20,21,22,23,24]. Unoh et al. discovered a noncovalent oral SARS-CoV-2 3CL^pro^ inhibitor (S-217622) used for treatment of COVID-19 [25]. Ketone-based covalent inhibitors of coronavirus 3CL^pro^ found by Hoffman et al. are potent inhibitors of CoV-2 3CLpro with suitable pharmaceutical properties warranting further development as an intravenous treatment toward COVID-19 [26]. Liu et al. identified a compound, termed coronastat, that was considered as a new candidate for a small molecule protease inhibitor for treating COVID-19 [27]. Noncovalent inhibitor (WU-04) of SARS-CoV-2 3CL^pro^ reported by Hou et al. effectively blocks SARS-CoV-2 replications in human cells with EC50 values in the 10-nM range [28], being regarded as a promising drug candidate for coronavirus treatment. Binding of inhibitors in the catalytic active sites leads to conformational responses of 3CL^pro^ [29,30]. In addition, ligand binding also causes a ligand size-dependent conformational change to the E-loop and linker of 3CL^pro^, further stabilizing the C-loop through hydrogen bonding interactions between the C-loop and E-loop. Therefore, it is highly requisite to probe inhibitor-3CL^pro^ interaction modes for further understanding the target roles of 3CL^pro^.

Molecular dynamics (MD) simulations [31,32,33,34,35], together with free energy analyses [36,37,38,39,40,41] and correlation network analysis, can be applied to probe the effect of inhibitor binding on conformational changes, network communication [42] and free energy profiles of targets. Conformations of proteins sampled by conventional MD (cMD) simulations are possibly trapped within an energy minima space because of the high energy barrier in simulation systems. Recently, to overcome this limitation, Gaussian accelerated molecular dynamics (GaMD) simulations, proposed by Miao’s group [43,44,45], employ a harmonic boost potential to smooth the free energy barrier of biomolecule and obtain higher sampling efficiency of conformations than cMD simulation, which has been verified in multiple works [46,47,48,49,50,51]. More importantly, MD simulations integrated by binding free energy predictions have obtained successes in insights into inhibitor-3CL^pro^ binding mechanisms [52,53,54,55]. Kumar et al. applied molecular docking and MD simulations to decipher the structure–activity relationship of inhibitor-3CL^pro^ complexes and reported a novel compound used as a strong candidate for therapeutic discovery against COVID-19 [56]. MD simulations and the 3D-QSAR method were combined to design new dipeptide inhibitors of 3CL^pro^ and probe binding modes of compound M-5 to 3CL^pro^ [57]. Moritsugu and coworkers adopted MD simulations to explore binding/unbinding pathways of 8-residue peptide substrate to/from 3CL^pro^ and the results revealed molecular mechanism of how a highly flexible peptide fold into the bound form [58], which can aid drug design toward 3CL^pro^. Despite these successes, it is still highly essential to further decipher inhibitor-mediated influences on conformational alterations of 3CL^pro^ for development of anti-COVID-19 drug.

With expectation of probing inhibitor-mediated conformational responses of 3CP^pro^, three inhibitors 7YY, 7XB and Y6G were selected for this study and their structures were depicted at Figure 1C,D. It is observed that two inhibitors 7YY and 7XB share similar topology structures but their IC50 values are 0.013 and 8.6 μM, respectively [25], showing highly different binding ability to 3CL^pro^. Inhibition ability of Y6G on the activity of 3CL^pro^ is 0.148 μM [59]. Similar molecular structures lead to different binding ability to 3CL^pro^ and their related molecular mechanism is interesting, which is reason why we selected these inhibitors. Thus, it is of importance to probe molecular mechanism underlying binding difference in inhibitors to 3CL^pro^ for design of 3CL^pro^ inhibitors toward treatment of COVID-19. To achieve our goal, GaMD simulations were performed to enhance conformation sampling, normal mode analysis (NMA) [60] and principal component analysis (PCA) [61,62,63] were conducted to probe conformational alterations and correlation network analysis (CNA) was adopted to decode changes in network communications induced by inhibitor binding [64,65,66]. In addition, molecular mechanics generalized Born surface area (MM-GBSA) [67,68,69] and quantum mechanics/MM-GBSA (QM/MM-GBSA) methods [70,71] were employed to estimate binding free energies of 7YY, 7XB and Y6G to 3CL^pro^ and comparatively uncover the corresponding free energy basis.

## 2. Results

### 2.1. Structural Properties of 3CLpro Revealed by GaMD Simulations

**Figure 2:**
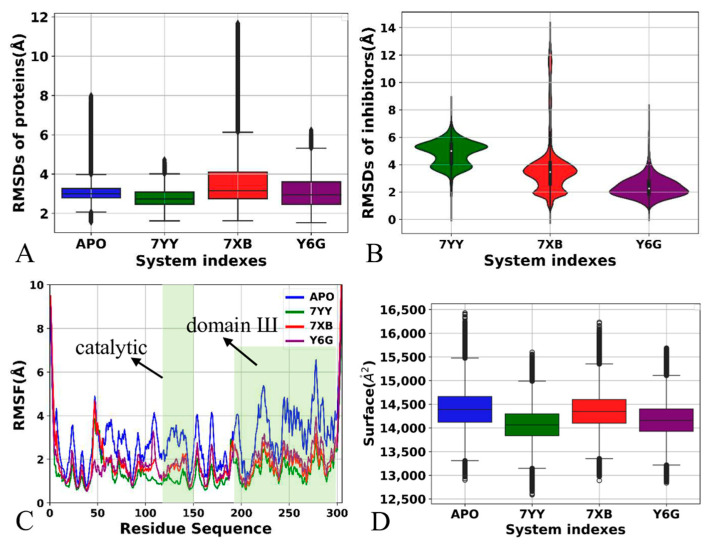
Structure properties captured by GaMD simulations: (**A**) RMSDs of backbone atoms from 3CL^pro^, in which the blue, green, red and purple, respectively, represent the APO 3CL^pro^, 7YY-, 7XB and Y6G-bound 3CL^pro^, (**B**) RMSDs of all heavy atoms from three inhibitors, from which the green, red and purple indicate inhibitors 7YY, 7XB and Y6G individually, (**C**) RMSFs of 3CL^pro^ calculated by using the Cα atoms and (**D**) SASAs of 3CL^pro^. The horizontal lines were used to easily identify the amplitudes.

To reveal structural fluctuations of 3CL^pro^, root-mean-square deviations (RMSDs) of backbone atoms from the single integrated GaMD trajectory, including sim1, sim2 and sim3, were calculated by referencing to the initially optimized structure (Figure S1A). The structures of 3CL^pro^ in four simulation systems fluctuate in a range of 1.49–11.69 Å. As shown in Figure S1A, the APO form shows high structural fluctuations in sim3 while 7XB-bound 3CL^pro^ has big structural fluctuation in sim1, sim2 and sim3, implying different subspace of conformation sampling are detected by GaMD simulations. The averaged RMSDs of APO, 7YY-, 7XB and Y6G-bound 3CL^pro^ are 2.99, 2.69, 3.14 and 2.93 Å, respectively, implying that binding of 7YY reduces structural fluctuation of 3CL^pro^ while binding of 7XB highly increases structural fluctuation (Figure 2A). The RMSDs of heavy atoms for 7YY, 7XB and Y6G were also estimated relative to their initially optimized structures using the single integrated GaMD trajectory (Figure S1B). It is found that the structures of 7YY and Y6G display a stable structural fluctuation, only 7XB greatly deviates from its initially optimized conformation at the end of sim2, which corresponds to the big structural fluctuation of 3CL^pro^ at the end of the sim2. The RMSDs of 7YY and Y6G are populated at 5.02 and 2.30 Å, respectively, but the RMSD of 7XB is distributed at 3.41 and 11.24 Å (Figure 2B), suggesting that the structural stability of 7YY and Y6G in binding pocket of 3CL^pro^ is higher than that of 7XB.

To understand the effect of inhibitor binding on structural flexibility of 3CLpro, root-mean-square-fluctuations (RMSFs) were computed with the coordinates of the Cα atoms in 3CL^pro^ (Figure 2C). Meanwhile, the difference in RMSFs between inhibitor-BOUND 3CL^pro^ and the APO one was also computed with the equation $\Delta \mathrm{RMSF}=RMSF_{bound}-RMSF_{APO}$, shown in Figure S2. Overall, except for the N-terminal and C-terminal regions, the binding of 7YY, 7XB and Y6G deceases the RMSFs of 3CL^pro^, making the structure of 3CL^pro^ more rigid compared to the APO 3CL^pro^ (Figure S2). It is worth noting that the presence of three inhibitors in binding pocket highly reduces the RMSF of the catalytic sites of 3CL^pro^ relative to the APO 3CLpro (Figure 2C), which may produce significant impacts on the catalytic activity of 3CL^pro^. Although binding sites of three inhibitors are distal from the domain III (the C-terminal), binding of 7YY, 7XB and Y6G still weakens the structural flexibility of the domain III (Figure 1A, Figure 2C and Figure S2), which reflects conformation response or network communications between them.

To probe the influences of inhibitor binding on hydrophily of 3CL^pro^, solvent accessible surface area (SASA) of 3CL^pro^ were calculated based on the single integrated GaMD trajectory (Figure S3 and Figure 2D). The SASAs of 3CL^pro^ in four systems fluctuate from 12,590 to 16,440 Å^2^ (Figure S3). The averaged SASAs of the APO 3CL^pro^ and 7YY-, 7XB- and Y6G-bound 3CL^pro^ are 14,398, 14,078, 14,338 and 14,148 Å^2^, individually (Figure 2D). By comparison with the APO 3CL^pro^, the SASAs of 7YY-, 7XB- and Y6G-bound 3CL^pro^ are decreased by ~320, 60 and 250 Å^2^, respectively, thus the binding of 7YY, 7XB and Y6G decrease the contacting extents of 3CL^pro^ with solvents and hydrophily of 3CL^pro^, which in turn affects the activity of 3CL^pro^.

Based on the current analyses, three findings are observed: (1) binding of 7YY, 7XB and Y6G exerts different impacts on structural fluctuations of 3CL^pro^, furthermore the structural stability of 7YY and Y6G in binding pocket of 3CL^pro^ is higher than that of 7XB, (2) the structure of 3CL^pro^ becomes more rigid due to binding of 7YY, 7XB and Y6G, in particular the catalytic sites, which certainly impacts the activity of 3CP^pro^ and (3) the hydrophily of 3CL^pro^ was weakened by binding of three inhibitors, reflecting the conformational changes of 3CL^pro^ and the effect on the activity of 3CL^pro^. Our current findings are fundamentally supported by the previous work [72,73].

### 2.2. Principal Component Analysis and Free Energy Profiles of 3CL^pro^

**Figure 3:**
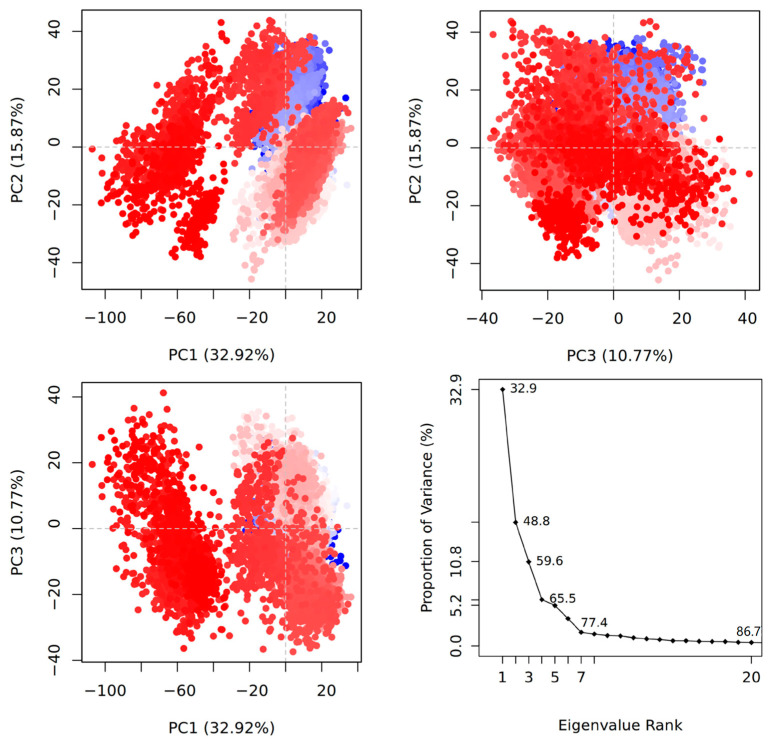
Information alterations of APO 3CL^pro^ captured by principal component analysis. The first three principal components PC1–PC3 account for fluctuating regions with 59.6% of overall fluctuations. In this figure, the blue region describes the most significant movements, while the red region reflects the least flexible motions. The transformation from the blue to the red implies conformation transition and the pale red or pale blue regions embody intermediate states during conformation transition.

To reveal conformational changes of 3CL^pro^ caused by binding of 7YY, 7XB and Y6G, PCA was performed on GaMD trajectories through the Bio3D package to produce eigenvectors and eigenvalues. The projections (PC1, PC2 and PC3) of the single integrated GaMD trajectory onto the first three eigenvectors and the evolutions of eigenvalues over the first twenty eigenvector indexes were depicted over the separate eigenvector indexes of the first 20 modes of motions (Figure 3 and Figures S4–S6). The PCA of four 3CL^pro^-related systems shows conformational changes of 3CL^pro^, from which the blue region describes the most significant movements, the pale red or pale blue regions embody intermediate motions and the red region reflects the least flexible motions. On the whole, movements of 3CL^pro^ were scaled by eigenvectors, which are mostly represented by the top-seven eigenvectors in the four current systems. The first seven eigenvectors, respectively, account for dominant movements with eigenvalues of 32.9–77.4, 25.1–70.6, 60–89 and 41.6–83.2% for the APO, 7YY-, 7XB- and Y6G-bound 3CL^pro^ (Figure 3 and Figures S4–S6). However, the remaining eigenvectors have lower eigenvalues, which corresponds to the local and rigid movements. For the APO 3CL^pro^, the projection PC1 accounts for the 32.9% of total motions for 3CL^pro^, PC2 accounts for 15.87% and PC3 has 10.77% proportion of total movements (Figure 3). This result indicates that the projection PC1 shows greater structural fluctuation relative to PC2 and PC3. By comparison, the PCA results of 7YY-, 7XB- and Y6G-bound 3CL^pro^ show similar behavior to the APO one (Figures S4–S6). Based on the cumulative variance of the complexes’ mobility described by the first seven principal components, the binding of 7YY weakens the mobility of 3CL^pro^ compared to the APO 3CL^pro^ (Figure S4) but the associations of 7XB and Y6G strengthens the mobility of 3CL^pro^ (Figures S5 and S6). Overall, the weaker variability of PC3 relative to the APO 3CL^pro^ implies the highly stabilized inhibitor-3CL^pro^ binding and a compact structure when referencing to the PC1 and PC2 variability (Figure 3 and Figures S4–S6).

**Figure 4:**
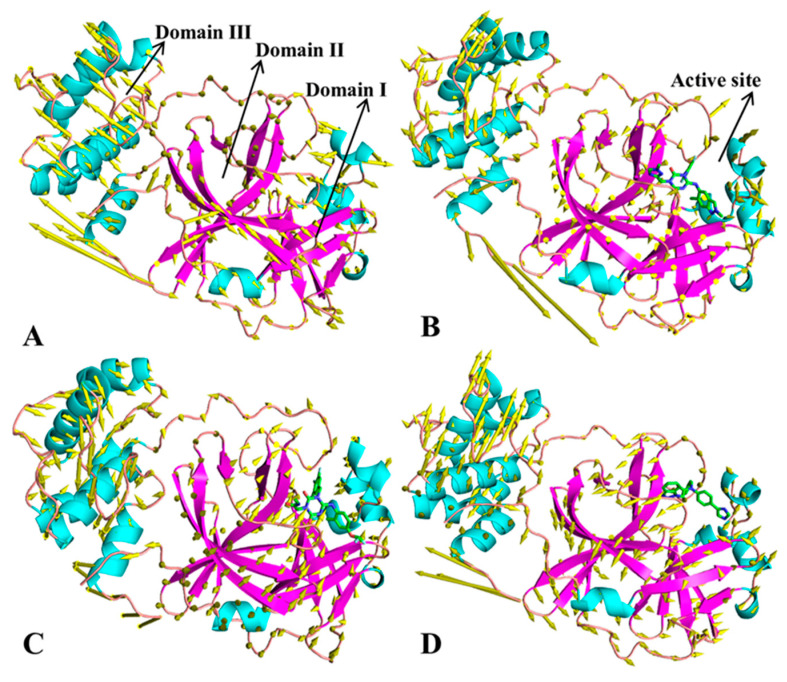
Concerted motions of structural domains in 3CL^pro^ reflected by the first eigenvector: (**A**) APO 3CL^pro^, (**B**) 7YY-bound 3CL^pro^, (**C**) 7XB-bound 3CL^pro^ and (**D**) Y6G-bound 3CL^pro^.

To identify inhibitor-mediated effects on dynamics behavior, the first eigenvectors of the four systems were visualized (Figure 4). Apart from the high flexibility of the tail in the C-terminal, the structural domains of 3CL^pro^ show highly concerted motions. In the APO 3CL^pro^, the helixes and the loop (linker2), located near catalytic sites between domains I and II, have an inward fluctuation tendency (Figure 4A and Figure S7A), but these two regions display an outward tendency in the bound states of three inhibitors (Figure 4B–D and Figure S7B–D). This change in dynamics behavior of catalytic sites certainly affects the activity of 3CL^pro^. Although binding sites of 7YY, 7XB and Y6G are distal from domain III, their binding changes fluctuation tendency of domain III compared to the APO 3CL^pro^ (Figure 4). Despite this, how inhibitor binding affects dynamics behavior of domain III is unclear; it is requisite to further probe network communications between structure domains. In addition, the binding of 7XB leads to disorder of fluctuation tendency in domain III by referencing to the APO 3CL^pro^.

**Figure 5:**
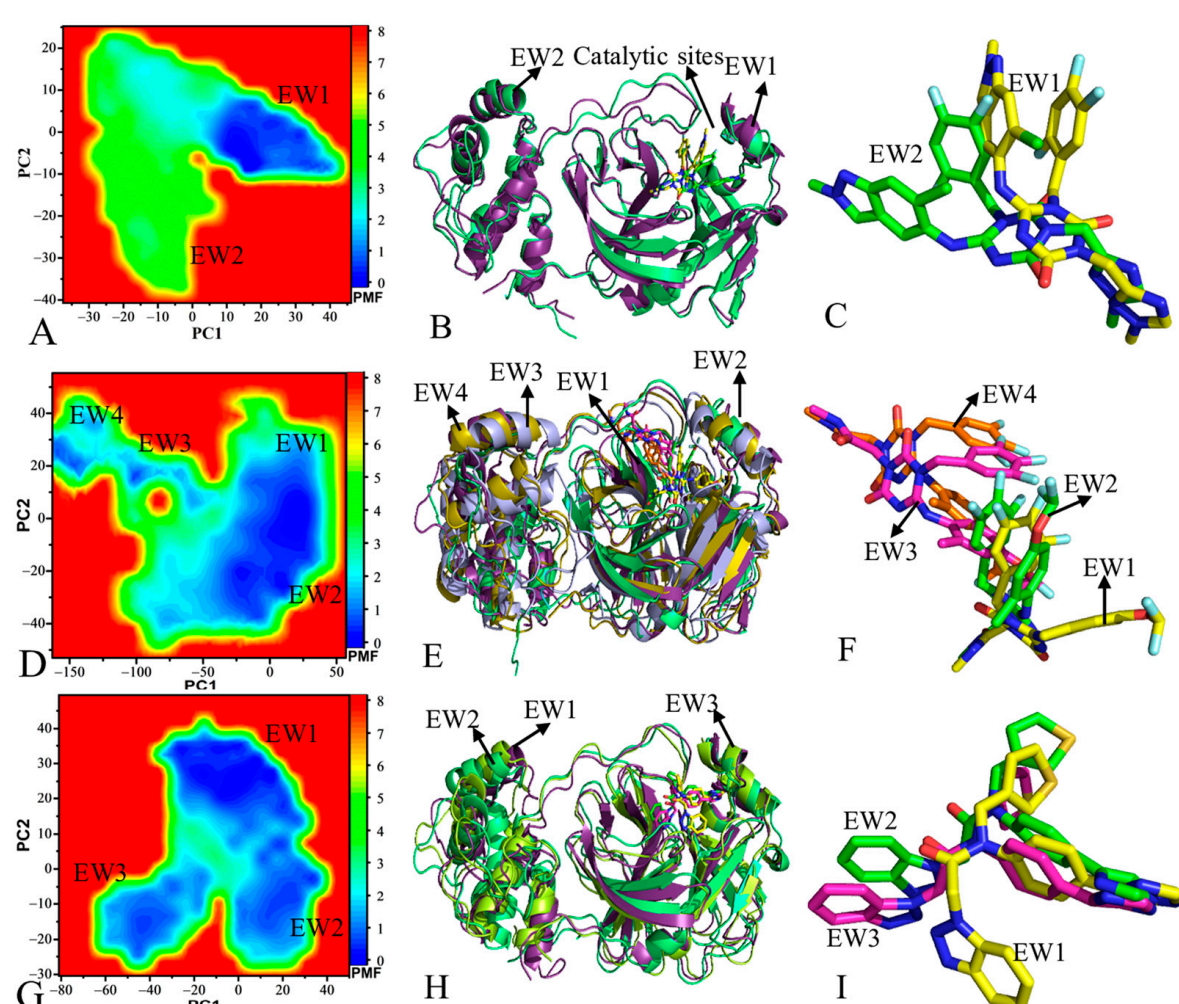
Free energy profiles and representative structures of three inhibitor-3CL^pro^ complexes: (**A**), (**D**) and (**G**) corresponding to free energy landscapes of 7YY-, 7XB- and Y6G-bound 3CL^pro^, respectively, (**B**), (**E**) and (**H**) indicating superimposition of representative structures for 7YY-, 7XB- and Y6G-bound 3CL^pro^, individually, and (**C**), (**F**) and (**I**) denoting structure alignment of 7YY, 7XB and Y6G falling into energy wells, separately. The potential of mean force (PMF) is scaled in kcal/mol.

To reveal the energy basis for conformational changes of 3CL^pro^, free energy landscapes (FELs) were constructed by using projections (PC1 and PC2) of the single integrated GaMD trajectory onto the first two eigenvectors as reaction coordinates (RCs) (Figure 5 and Figure S8). For the APO 3CL^pro^, four free energy wells (EW1-EW4) are captured by GaMD simulations (Figure S8A). The projection of the GaMD trajectory for the APO 3CL^pro^ onto the first eigenvector (PC1) fluctuates from −38.7 to 107.1 Å, while the projection onto the second eigenvector (PC2) is located at a range of −46.8–48.9 Å (Figure S8A). The superimposition of four representative structures falling into the EW1-EW4 indicates that the loops in the catalytic regions and domain III produce big deviations from each other (Figure S8B and Figure 1A). In the 7YY-bound 3CL^pro^, two main energy wells (EW1 and EW2) are identified through the entire GaMD simulation (Figure 5A). The projection of the single integrated GaMD trajectory for the 7YY-bound 3CL^pro^ onto the first eigenvector (PC1) falls into a fluctuation range of −37.4–49.9 Å while the projection onto the second eigenvector is situated at a range of −40.6 to 28.5 Å (Figure 5A), indicating that the binding of 7YY weakens mobility of 3CL^pro^ compared to the APO state. The alignment of two representative structures situated in the EW1 and EW2 suggests that the structures of 7YY-bound 3CL^pro^ do not generate obvious structural deviations (Figure 5B) but 7YY evidently deviates from each other between two representative structures (Figure 5C). As for the 7XB-bound 3CL^pro^, GaMD simulations detect four primary energy wells EW1-EW4 (Figure 5D). The projection PC1 for the 7XB-bound 3CL^pro^ fluctuates from −162.7 to 65.3 Å but the projection PC2 fluctuates from −54.3 to 60.8 Å (Figure 5D). By comparison with the APO 3CL^pro^, the binding of 7XB strengthens the mobility of 3CL^pro^. The superimposition of four representative structures for the 7XB-bound 3CL^pro^ located at the EW1-EW4 shows that the loops and α-helix in the catalytic regions and domain III yield great deviations from each other among the EW1-EW4 structures (Figure 5E). The structural alignment of 7XB in the EW1-EW4 indicates that 7XB has four different binding poses and obvious structural deviations (Figure 5F), which affects the binding of 7XB to 3CL^pro^. With respect to the Y6G-bound 3CL^pro^, three main energy wells (EW1-EW3) are recognized during the entire GaMD simulations (Figure 5G). The projection PC1 for the Y6G-bound 3CL^pro^ falls into a fluctuation range from −80.5 to 49.5 but the PC2 from −30.1 to 53.4 Å (Figure 5G). In contrast to the APO 3CL^pro^, the binding of Y6G weakens the mobility of 3CL^pro^. According to the superimposition of three representative structures for the Y6G-bound 3CL^pro^, the loops in domain III produce evident deviations (Figure 5H). Furthermore, three binding poses of Y6G also obviously deviate from each other (Figure 5I).

Based on the aforementioned analyses, the binding of the three inhibitors exerts key effects on dynamics behavior of 3CL^pro^: (1) binding of 7YY, 7XB and Y6G changes fluctuation tendency of 3CL^pro^ structure along the first eigenvector, (2) the presence of the three inhibitors affects free energy profiles and induces conformation rearrangement of 3CL^pro^ and (3) the binding of 7YY and Y6G weakens the mobility of 3CL^pro^ while the 7XB binding strengthens that of 3CL^pro^. Thus, the dynamics changes of 3CL^pro^ caused by inhibitor binding certainly influence the activity of 3CL^pro^. The study from Xiong et al. showed that the complex systems of 3CL^pro^ possess a more concentrated motion mode by comparison with APO 3CL^pro^ [72], fundamentally agreeing with our results. The results of MD simulations performed by Jawarkar et al. suggested that domain III produces positional variation relative to catalytic regions [74], being in basic consistence with our current findings.

### 2.3. Normal Mode Analysis and Correlation Network Analysis

**Figure 6:**
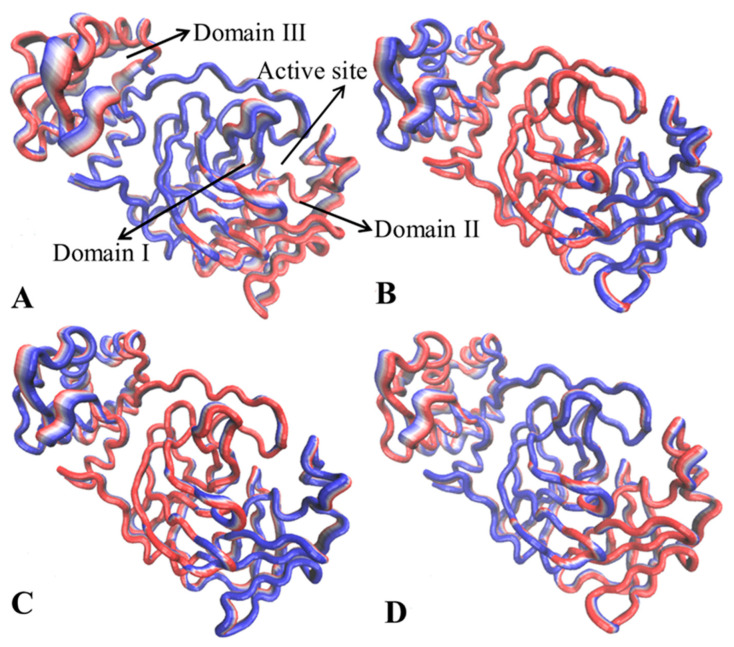
Conformational dynamics of key structure domains detected by principal component analysis: (**A**) APO 3CL^pro^; (**B**) 7YY-bound 3CL^pro^; (**C**) 7XB-bound 3CL^pro^; (**D**) Y6G-bound 3CL^pro^.

To further investigate conformational changes of 3CL^pro^ caused by inhibitor binding, NMA was performed using the Bio3D package on the crystal structures 7JVZ (https://www.rcsb.org/structure/7JVZ (12 January 2024)), 7VU6, 7VTH and 7LMF corresponding to APO, 7YY-, 7XB- and Y6G-bound 3CL^pro^, respectively, and the results of the most significant mode for four structures were visualized in Figure 6. The transformation from blue to red represents changes in conformations and flexibility. It is worth noting that the conformations of the loops and α-helix near the catalytic sites and domain III are mostly influenced by the binding of 7YY, 7XB and Y6G (Figure 1A and Figure 6). For APO 3CL^pro^, the structural domains near the catalytic sites, including the loops and α-helix, show high structural flexibility and conformational alterations (Figure 6A). Furthermore, the domain III of APO 3CL^pro^ also has great structural flexibility and mobility (Figure 6A). Compared to APO 3CL^pro^, binding of the three inhibitors abates structural flexibility and mobility of the catalytic regions (domains I and II) and tends to make these regions more rigid (Figure 6C,D). Meanwhile, the structural flexibility and mobility of domain III are also weakened by the presence of the three inhibitors relative to APO 3CL^pro^ (Figure 6C,D). The alteration in structural flexibility and mobility in turn yields significant impacts on the catalytic activity of 3CL^pro^. The analysis of dynamics cross-correlation maps (DCCMs) performed by the Bio3D package was shown in Figure S9. For APO 3CL^pro^, the region R1 located between domain I and II generates strongly positive correlation motions (indicated by the cyan) and the region R2 reflects the strongly anti-correlated motions (indicated by the violet) of the catalytic sites (residues 100–148) relative to residues 50–85 (Figure S9A). By referencing to APO 3CL^pro^, the binding of 7YY obviously weakens the correlated motions occurring at the regions R1 and R2 (Figure S9B) while the binding of 7XB evidently strengthens correlated movements of these two regions (Figure S9C). Differently, the binding of Y6G only yields slight impacts on correlated motions of the regions R1 and R2 compared to APO 3CL^pro^ (Figure S9D).

**Figure 7:**
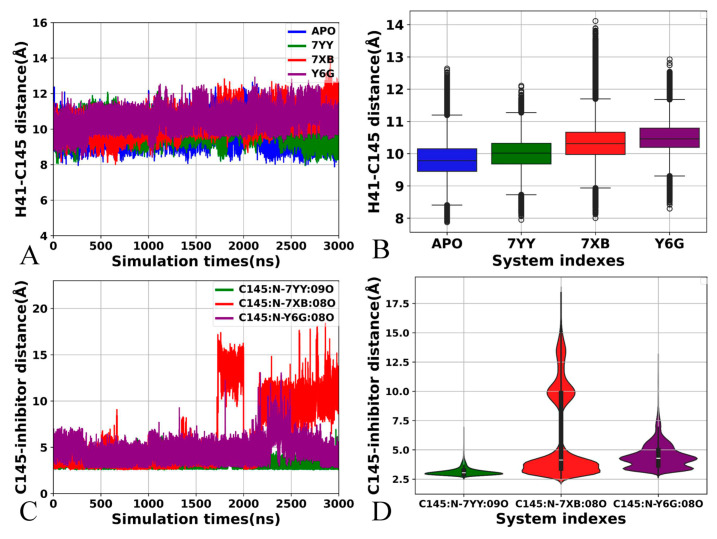
Distances involved in catalytic residue C145: (**A**) the function of distances between the Cα atoms of residues C145 and H41 as the simulation time, (**B**) statistical distributions of the H41-C145 distance, in which the blue, green, red and purple, respectively, represent the APO 3CL^pro^, 7YY-, 7XB and Y6G-bound 3CL^pro^, (**C**) the function of distance between C145 and inhibitors as the simulation time and (**D**) statistical distribution of the distances between C145 and inhibitors, from which the green, red and purple indicate the distances corresponding to inhibitors 7YY, 7XB and Y6G individually. The horizontal lines were used to easily identify the amplitudes.

To clarify the changes in internal conformations of catalytic sites, the distance between the Cα atoms of the catalytic residue C145 and H41 was calculated using the CPPTRAJ program (Figure 7A,B). This distance fluctuates from 7.84 to 14.33 Å in our current systems (Figure 7A). It is also observed that this distance is populated at 9.76, 10.12, 10.28 and 10.47 Å in APO, 7YY-, 7XB- and Y6G-bound 3CL^pro^, individually (Figure 7B). Thus, binding of 7YY, 7XB and Y6G leads to the increase of 0.36, 0.52 and 0.71 Å in the distance between the Cα atoms of C145 and H41 compared to APO 3CL^pro^, which may slightly affect the catalytic activity of 3CL^pro^. In addition, the distances of the nitrogen atom (N) in C145 away from the oxygen atoms 09O in 7YY, 08O in 7XB and 08O in 6YG were also calculated with the CPPTRAJ program (Figure 7C,D). These distances between C145 and inhibitors fall into a fluctuation range from 2.47 to 18.49 Å and the distance between the nitrogen atom N of C145 and the oxygen atom 08O of 7XB shows high alterations (Figure 7C). These distances in the 7YY- and Y6G-bound 3CL^pro^ are distributed at 3.08 and 4.33 Å, respectively. The 7XB-bound 3CL^pro^ is populated at two peak values of 4.21 and 9.91 Å (Figure 7D). Therefore, the alterations in the distances between catalytic residue C145 and inhibitors can yield key influences on inhibitor-3CL^pro^ binding.

**Figure 8:**
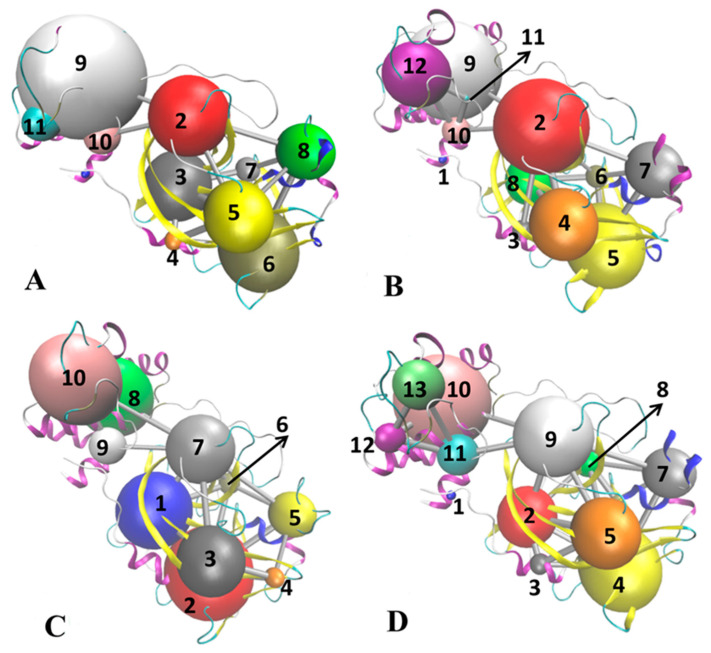
Changes in communication networks caused by inhibitor binding: (**A**) APO 3CL^pro^, (**B**) 7YY-bound 3CL^pro^, (**C**) 7XB-bound 3CL^pro^ and (**D**) Y6G-bound 3CL^pro^. In this figure, balls are used to represent nodes and sticks are adopted to indicate edges describing communications between different nodes.

The previous analyses suggest that binding of inhibitors in catalytic sites changes structural flexibility of domain III distal from the binding sites, but the corresponding mechanism is unclear. To solve this issue, the CNA was performed on the correlation matrix $C_{ij}$ calculated by using conformational ensembles, in which the cutoff value of $C_{ij}$ is set to 0.4. The results from the CNA were mapped onto the initially optimized structures of four current systems (Figure 8) and the details were displayed in Figure S10. As shown in Figure 8 and Figure S10, correlation networks are constructed using the CNA, in which nodes represent residues of 3CL^pro^ and edges are weighted by the strength of their respective correlation values. This method has obtained successes in insights into allosteric couplings in a range of systems [75,76,77]. For APO 3CL^pro^, although the cluster results reveal 11 nodes, six mainly consistently correlated protein sectors (or community groups, including nodes 2, 3, 5, 6, 8 and 9) play key roles in conformation responses, in which nodes 2 and 3 are located at domain II, 5, 6 and 8 at domain I and 9 at domain III (Figure 8A and Figure S10A,B). It is observed that the node 2 (red) in domain II builds a bridge to realize network communication between two distal domains I and III of APO 3CL^pro^ through nodes 9 and 10 in domain III (Figure 8A and Figure S10B). Among 12 nodes of 7YY-bound 3CL^pro^ recognized by the CNA, seven community groups involving nodes 2, 4, 5, 7, 8, 9 and 12 are responsible for conformational responses between structural domains (Figure 8B and Figure S10C,D), of which nodes 2 and 8 are in domain II, nodes 4, 5 and 7 in domain I and nodes 9 and 12 in domain III. The conformational alterations near catalytic sites (nodes 4, 5 and 7) caused by the binding of 7YY are transferred into domain II (node 2) and then conformational responses in domain II are transferred into domain III (Figure 8B and Figure S10D), which realizes the network communication between domains I and III. In 7XB-bound 3CL^pro^, the CNA recognizes 10 nodes, of which seven nodes 1, 2, 3, 5, 7, 8 and 10 play key roles in conformational responses (Figure 8C and Figure S10E,F). The conformational changes next to catalytic sites (nodes 2, 5 and 3 in domain I) induced by the binding of 7XB are transferred into domain III through nodes 1, 7, 8, 9, 10 (Figure 8C and Figure S10F). As for Y6G-bound 3CL^pro^, 13 nodes are identified by the CNA (Figure 8D and Figure S10G,H), of which 8 consistently correlated protein sectors (nodes 2, 4, 5, 7, 9, 10, 11 and 13) take part in transferring of conformational responses. It is observed that conformational alterations near catalytic sites caused by the Y6G binding are transferred to node 9 in domain II and then also transferred to nodes 10 and 11 in domain III (Figure 8D and Figure S10H).

Based on the above analyses, binding of inhibitors mediates the effect on structural flexibility, mobility and network communications of 3CL^pro^: (1) the presence of inhibitors not only alters conformational flexibility of catalytic regions but also weakens conformational mobility of domain III, (2) the distances between catalytic residue C145 and inhibitors yield great changes, implying the alterations in binding ability and (3) a node in domain II forms a bridge transferring conformation responses of catalytic sites caused by binding of inhibitors into domain III. Domain III is an α-helical domain shown to be critical for dimerization, thus conformation responses to the effect of inhibitor binding on catalytic regions certainly disturb the activity of 3CL^pro^ [59]. The crux of the scalable all-atom MD simulations consummated in explicit solvent media of Samanta et al. captured the structural plasticity of 3CL^pro^ induced by the binding of remdesivir analogs [78]. MD exploration of Albani et al. indicated that ligand binding destabilizes the catalytically active conformation of the H41/C145 dyad [30], which is agrees basically with our current findings.

### 2.4. Calculations of MM-GBSA and QM/MM-GBSA

To reveal factors affecting binding of inhibitors to 3CL^pro^, the MM-GBSA and QM/MM-GBSA, methods were applied for calculating binding free energies of 7YY, 7XB and Y6G to 3CL^pro^. The results for MM-GBSA calculation are listed in Table 1 while the results for the QM/MM-GBSA calculation are in Table 2. Binding free energies of 7YY, 7XB and Y6G to 3CL^pro^ calculated with the MM/GBSA method are −14.66, −13.89 and −14.27 kcal/mol (Table 1), respectively, while those estimated using the QM/MM-GBSA method are −13.45, −11.68 and −11.83 kcal/mol, respectively (Table 2). The rank of the calculated binding free energies utilizing these two methods is in consistence with the one determined by experimental values, implying the reliability of our current binding free energy analyses. In addition, we also used MM-PBSA method to estimate binding free energies of three inhibitors to 3CL^pro^ (Table S1). Binding free energy of 7YY, 7XB and Y6G to 3CL^pro^ are −6.05, −1.17 and −4.78 kcal/mol, respectively. Therefore, the binding free energies of the three inhibitors predicted by the MM-PBSA method are much weaker than not only the experimental values but also the results calculated by MM-GBSA and MM/QM-GBSA. Based on this comparison, the MM-GBSA and MM/QM-GBSA methods were used to compute binding free energies.

In the MM-GBSA calculations, van der Waals interactions ($\Delta E_{vdW}$), electrostatic interactions ($\Delta E_{ele}$) and non-polar solvation free energies ($\Delta G_{esurf}$) provide favorable driving forces for binding of the three inhibitors (Table 1) while polar solvation free energies ($\Delta G_{egb}$) is an unfavorable factor for binding. These four terms form binding enthalpy (∆H) of inhibitors and the ∆H of 7YY, 7XB and Y6G to 3CL^pro^ are −41.71, −41.52 and −40.29 kcal/mol (Table 1), respectively. In QM/MM-GBSA calculations, van der Waals interactions ($\Delta E_{vdW}$), quantum mechanics energies ($\Delta G_{esurf}$) and non-polar solvation free energies ($\Delta G_{esurf}$) are responsible for most favorable forces during associations of inhibitors with 3CL^pro^ but polar solvation free energies ($\Delta G_{egb}$) screen the favorable forces (Table 2). These four components construct the ∆H of 7YY, 7XB and Y6G to 3CLpro and their strengths −40.5, −39.31 and −37.85 kcal/mol (Table 2), respectively. The entropy contributions (−T∆S) are unfavorable factors for inhibitor-3CL^pro^ binding and the binding entropies of 7YY, 7XB and Y6G to 3CL^pro^ are 27.05, 27.63 and 26.02 kcal/mol (Table 1 and Table 2), respectively.

To test the results of MM-GBSA and QM/MM-GBSA calculations, 7YY, 7XB and Y6G were docked into binding pocket of 3CL^pro^ to obtain their binding affinity (Table S2). The docked structures of the first twenty docking scores were depicted in Figure S11, in which the best binding poses of 7YY, 7XB and Y6G were aligned with those in three crystal structures 7VU6, 7VTH and 7LMF and their RMSDs are 0.96, 1.15 and 1.21 Å, respectively. Binding strength of three inhibitors scaled by the first six scoring is in the order 7XB < Y6G < 7YY, agreeing with not only the rank of binding free energies calculated by MM-GBSA and QM/MM-GBSA but also that determined by the experimental values. These results suggest that our MM-GBSA and QM/MM-GBSA calculations are reliable. Figure S11 indicates that 7YY, 7XB and Y6G have three, four and four binding sites, respectively. 7YY, 7XB and Y6G with the first six scoring are docked at the site 1, which is in consistence with binding site from three crystal structures 7VU6, 7VTH and 7LMF. The three other binding sites (Sites 2–4) may imply possible allosteric binding sites.

Based on the aforementioned information, binding free energies derived from MM-GBSA, QM/MM-GBSA, MM-PBSA and molecular docking are in good consistence with the experimental values in the order, which support our free energy analyses. More interestingly, van der Waals interactions primarily drive the binding of three current inhibitors to 3CL^pro^. This implies that van der Waals interactions should be paid special attentions in future development of non-covalent bond inhibitors toward 3CL^pro^. The work of Samanta et al. not only found that the polar solvation energy contributes unfavorably to the binding free energy and annihilates the contribution of electrostatic interaction but also verified that the van der Waals interactions with the active site residues show the augmentation of inhibitory efficacy of the remdesivir analog [78]. According to Figure 1C,D, 7XB and 7YY share highly similar structures to each other. The G2 group and the alkyl groups of 7XB are replaced by the G1 group and five-membered rings in 7YY, respectively, which leads to an increase in binding ability from 7YY to 3CL^pro^, relative to 7XB (Table 1 and Table 2). Thus, modification in molecular scaffold can be applied for design of potent 3CL^pro^ inhibitors.

### 2.5. Target Sites to 3CL^pro^ Identified by Residue-Based Free Energy Decomposition

**Figure 9:**
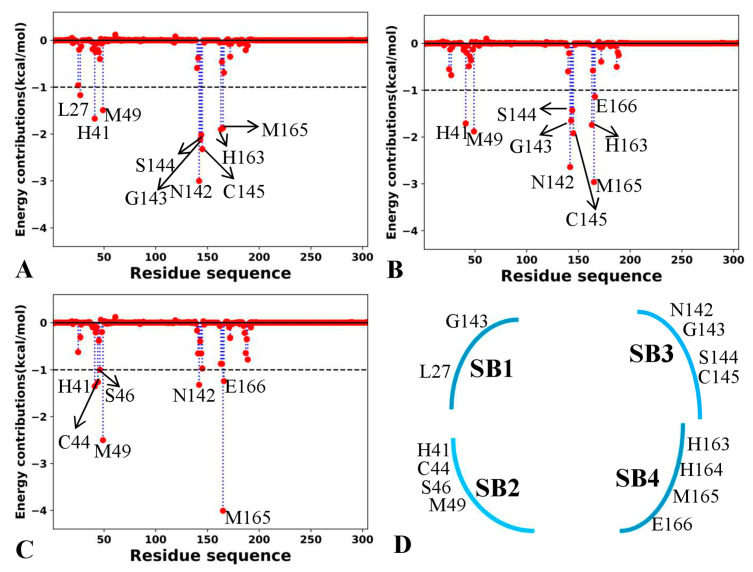
Interaction spectrum of inhibitors with separate residues from 3CL^pro^: (**A**) 7YY, (**B**) 7XB, (**C**) Y6G and (**D**) key residues belonging to different subpockets of 3CL^pro^.

**Figure 10:**
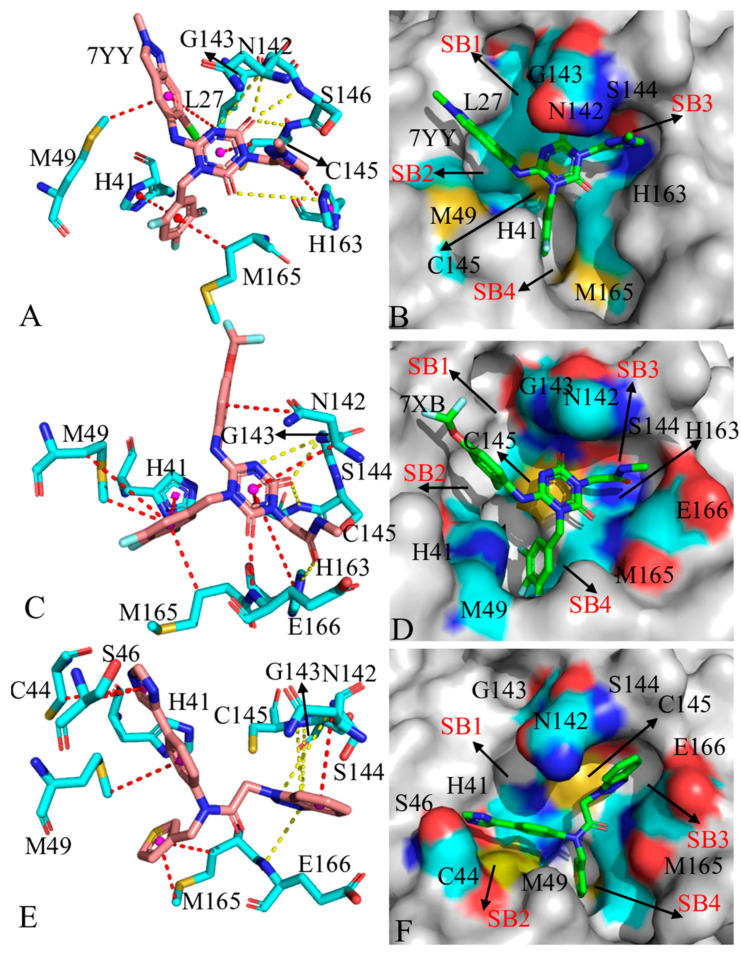
Geometric information for interactions of inhibitors with key residues and binding sub pockets: (**A**) 7YY-residue interactions, (**B**) binding subpockets of 7YY to 3CL^pro^, (**C**) 7XB-residue interactions, (**D**) binding subpockets of 7XB to 3CL^pro^, (**E**) Y6G-residue interactions and (**F**) binding subpockets of Y6G to 3CL^pro^. In this figure, inhibitors and key residues were displayed in stick modes while subpockets were shown in surface styles. The red dashed lines indicate hydrophobic interactions while yellow ones represent hydrogen bonding interactions.

Identification of target sites for proteins is critical for drug design. To probe this issue, residue-based free energy decomposition was performed to recognize hot interaction spot of residues in 3CL^pro^ with the three inhibitors (Figure 9A–C). Key residues forming four subpockets of 3CL^pro^ were displayed in Figure 9D. Hydrogen bonding interactions (HBIs) between 3CL^pro^ and inhibitors were dissected with the CPPTRAJ program (Table S3). To clarify which factors drive inhibitor-residue interactions, free energy contributions of significant residues were further decomposed into van der Waals interactions of backbones ($\Delta B_{vdW}$), side chains ($\Delta S_{vdW}$) and total ($\Delta T_{vdW}$), electrostatics interactions of backbones ($\Delta B_{ele}$), side chains ($\Delta S_{ele}$) and total ($\Delta T_{ele}$) and polar solvation free energies of backbones ($\Delta B_{gb}$), side chains ($\Delta S_{gb}$) and total ($\Delta T_{gb}$), which were provided in Table S4. The information of subpockets for 3CL^pro^ and key residues was depicted in Figure 10.

In 7YY-bound 3CL^pro^, nine residues provide energy contributions stronger than 1.0 kcal/mol, including L27, H41, M49, N142, G143, S144, C145, H163 and M165 (Figure 9A). The alkyls and CH groups of L27, M49, S144, C145 and M165 are next to the hydrophobic rings of 7YY (Figure 10A), thus the CH-π interactions are easy to be formed between them. S144 and C145 also forms hydrogen bonds (7YY-O09⋯C145-N-H) and (7YY-O09⋯S144-N-H) with an occupancy of 87.48 and 46.64% (Table S3), respectively. The interaction energies of L27, M49, S144, C145 and M165 with 7YY are −1.17, −1.49, −2.02, −2.32 and −1.87 kca/mol (Figure 9A and Table S4). The energy contributions of L27, M49 and M165 primarily stem from van der Waals interactions of their side chains (Table S4), implying that their side chains play significant roles in interactions with 7YY. The van der Waals interactions in the side chain and electrostatic interactions in the backbone of S144 and C145 provide most favorable contributions for the binding of 7YY (Table S4), in which the electrostatic interactions in their backbones agree well with the hydrogen bond 7YY-O09⋯C145-N-H and 7YY-O09⋯S144-N-H (Tables S3 and S4). The hydrophobic rings of H41 and H163 are situated near those of 7YY (Figure 10A), which tend to yield the π-π interactions between them. In addition, the side chain of H163 produces a HBI (7YY-O04⋯H163-NE2-HE2) with 7YY and its occupancy is 73.68% (Table S3 and Figure 10A). As a result, H41 and H163 provide energy contributions of −1.67 and −1.9 kcal/mol for the binding of 7YY (Figure 9A and Table S4). As shown in Table S4, the interaction energy of H41 mostly comes from the van der Waals interaction of its side chain. Differently, the interaction energy of H163 originates from not only the van der Waals interactions of its backbone but also the electrostatic interaction of its side chain (Table S4). The electrostatic interaction of the side chain in H163 is in agreement with the hydrogen bond 7YY-O04⋯H163-NE2-HE2 (Table S3 and Figure 10A). In addition, G143 forms two hydrogen bonds with 7YY, namely 7YY-N10⋯G143-N-H and 7YY-O09⋯G143-N-H with the occupancy of 60.29 and 57.36% (Table S3). Thus, G143 provides an energy contribution of −2.12 kcal/mol, which mostly comes from the electrostatic interactions of its backbone (Figure 9A and Table S4). It is noted that electrostatic interactions of residues with inhibitors are mostly screened by the polar solvation free energies of the side chains and backbones (Table S4). According to Figure 10B, residues L27 and G143 form the subpocket SB1, H41 and M49 form the SB2, N142, S144, C145 and H163 form SB3 and H41, H163 and M165 form the SB4. It is observed that the groups of 7YY reach into different subpockets (Figure 10B), which leads to interactions with the subpockets.

In the case of 7XB-bound 3CL^pro^, nine residues generate interactions stronger than 1.0 kcal/mol with 7XB (Figure 9B) including H41, M49, N142, G143, S144, C145, H163, M165 and E166. Similarly to 7YY-bound 3CL^pro^, the alkyls and CH groups of M49, N142, S144, C145, M165 and E166 are adjacent to the hydrophobic rings and alkyl of 7XB (Figure 10C). Thus, M49, N142, S144, C145 and M165 produce the CH-π interactions with 7XB while E166 generates the CH-CH interactions with 7XB (Figure 10C). In addition, C145 and E166 forms two hydrogen bonds, namely 7XB-O08⋯C145-N-H and 7XB-O35⋯E166-N-H, with the occupancy of 42.31 and 77.65%, respectively (Table S3 and Figure 10C). On the whole, M49, N142, S144, C145, M165 and E166 contribute interaction energies of −1.88, −2.64, −1.43, −1.92, −2.96 and −1.14 kcal/mol to the binding of 7XB (Figure 9B and Table S4). The interaction energy of M49 mainly arises from the van der Waals interaction of its side chain while that of M165 is mostly provided by the van der Waals interactions of its side chain and backbone (Table S4). The interaction energies of N142, S144, C145 and E166 originate from not only the van der Waals interactions of their side chains and backbones but also electrostatic interactions of their side chains and backbones (Table S4). The hydrophobic rings of H41 and H163 are situated near that of 7XB, thus they are easy to form the π-π interactions (Figure 10C). Meanwhile, H163 also produces a HBI with 7XB, namely 7XB-O04⋯H163-NE2-HE2, with an occupancy of 39.29%. Overall, H41 and H163 provide energy contributions of −1.71 and −1.74 kcal/mol for the binding of 7XB, respectively (Figure 9B and Table S4). The interaction energy of H41 primarily comes from the van der Waals interaction of its side chain and electrostatic interaction of its backbone while that of H163 is mainly contributed by the van der Waals interactions and electrostatic interactions of its sidechain (Table S4). In addition, G143 forms two hydrogen bonds with 7XB, including 7XB-O08⋯G143-N-H and 7XB-O09…G143-N-H with the occupancy of 46.77 and 16.32%, which extremely agrees with the electrostatic interactions of its backbone with 7XB (Tables S3 and S4). It is observed that electrostatic interactions of residues with inhibitors are counteracted by the polar solvation free energies of the side chains and backbones (Table S4), which partially weakens inhibitor-residue interactions. According to Figure 10D, G143 takes part in the formation of the subpocket SB1, H41 and M49 build the SB2, N142, S144, C145 and H163 construct the SB3 and H163, M165 and E166 form the SB4. It is found that the group of 7XB does not reach into the SB1 (Figure 10D), which makes the binding ability of 7XB weaker than 7YY. The experimental work of Unoh et al. also revealed that residues G143, C145, H163 and E166 form conserved hydrogen bonds with inhibitors [25]. Similar hydrogen bonds between 3CL^pro^ and inhibitors were also recognized in the X-ray Structures determined by Han and their coauthors [59]. These two experimental works verified our finding.

As for the Y6G-bound 3CL^pro^, seven residues H41, C44, S46, M49, N142, M165 and E166 are involved in interactions stronger than 1.0 kcal/mol with Y6G (Figure 9C). The alkyls or CH groups of C44, S46, M49, N142, M165 and E166 are located near the hydrophobic rings of Y6G, which tend to form the CH-π interactions between them (Figure 10E). Additionally, E166 forms a hydrogen bond (Y6G-O01⋯E166-N-H) with Y6G and its occupancy is 79.97% (Figure 10E and Table S3). On the whole, C44, S46, M49, M165 and E166 provide energy contributions of −1.26, −1.0, −2.5, −1.32, −4.01 and −1.24 kcal/mol for the binding of Y6G, separately (Figure 9C and Table S4). The interactions energies of M49, N142 and M165 mainly stem from the van der Waals interaction of their side chain (Table S4). The interaction energy of C44 originates from not only the van der Waals interaction of its side chain but also the electrostatic interaction of its backbone. The interaction energies of S46 and E166 are mostly contributed by the van der Waals interaction of their side chains and backbones (Table S4). The hydrophobic ring of H41 is next to that of Y6G and tends to yield the π-π interaction between them, which provides an energy contribution of −1.35 kcal/mol (Figure 9C and Table S4). This interaction energy comes from not only the van der Waals interactions of the side chain in H41 but also electrostatic interactions of the side chain and backbone in H41 (Table S4). It is also found that the polar solvation free energies of the side chains and backbones from all key residues highly screen favorable electrostatic interactions between residues and inhibitors (Table S4). In addition, G143 forms two hydrogen bonds (Y6G-N11⋯G143-N-H and Y6G-N12⋯G143-N-H) while C145 and S146, respectively, form a hydrogen bond (Y6G-N11⋯C145-N-H and Y6G-N11⋯S146-H-H), but the occupancy of these hydrogen bonds is lower than 17.34% (Table S3), thus providing the weak contribution for the binding of Y6G. As shown in Figure 10F, N142 and G143 are parts of the subpocket SB1, H41, C44, S46 and M49 form the SB2, N142, C145 and E166 build the SB3 as well as M49 and M165 construct the SB4. Unfortunately, no group of Y6G extends into the SB1.

Through the above information, it is found that the CH-π and π-π interactions together with HBIs play vital roles in inhibitor-3CLpro binding. Residues L27, H41, C44, S46, M49, N142, G143, S144, C145, H163, H164, M165 and E166 are identified as hot interaction spots, in which L27 and G143 belong to the SB1, H41, C44, S46 and M49 correspond to the SB2, N142, G143, S144 and C145 form the SB3 and H163, H164, M165 and M166 take part in the construction of SB4 (Figure 9D). These residues can be used as efficient targets of drug design for the treatment of COVID-19. The work of Han et al. revealed that C44, S46, M49, C145, H163, M165 and E166 play key roles in binding of inhibitors to 3CL^pro^ [59], supporting our current results. The study from Unoh et al. verified that H41, M49, G143, C145, H163 and E166 are responsible for most binding forces of inhibitors to 3CL^pro^ [25], basically agreeing with our work. Therefore, key residues identified by our study can be used as efficient targets to design clinically available inhibitors toward treatment of COVID-19.

## 3. Theory and Methods

### 3.1. Setup of Simulation Systems

The initial atomic coordinates of 7YY-, 7XB- and Y6G-bound 3CL^pro^ were derived from protein data bank (PDB) and their entries were 7VU6, 7VTH [25] and 7LMF [59], respectively. The APO 3CL^pro^ without inhibitor binding was obtained by deleting 7YY from 7VU6. Partial residues in the crystal structure 7VTH are missing, thus the program Modeller9.25 [79] was used to repair it into a complete structure. Due to difference in residue numbers of three complex structures, residues 1–306 were adopted to construct our simulation systems. Crystal water molecules were kept in the starting model. The missing hydrogen atoms in three crystal structures were bonded onto their corresponding heavy atoms with the Leap module [80] in Amber 22. The protonation states of 3CL^pro^ were examined using the program H++3.0 [81] and rational protonation states were set. In our current treatment, H41 and H80 were protonated at the delta nitrogen atom and all other histidine residues are epsilon-protonated, which is favorable for substrate binding [82]. The force field parameters of 7YY, 7XB and Y6G were derived from the general Amber force field (GAFF2) [83,84]. The Austin Model 1 with bond charge correction (AM1-BCC) method [85] was applied to assign the atomic charges of three 3CL^pro^ inhibitors with the Antechamber tool [86] in Amber22 [87]. The Amber ff14SB forced field was employed to yield force field parameters of 3CL^pro^ [88]. An octahedral periodic box of water with a buffer of 10.0 Å was utilized to separately solve APO 3CL^pro^ and 7YY-, 7XB- and Y6G-bound 3CL^pro^, in which the parameters of water molecules were obtained from the TIP3P model [89]. Each 3CL^pro^-related system was neutralized using sodium ion (Na^+^) in a 0.15 M NaCl salt environment. The parameters of all ions involved in this work, including Na^+^, and Cl^−^ ions, were set using the parameters recorded in the works of Joung and Cheatham [90,91].

### 3.2. GaMD Simulations

To explore the effect of the state for the removed inhibitor on conformation of 3CL^pro^, GaMD simulations on the APO 3CL^pro^ formed by deleting the inhibitor and inhibitor-bound 3CL^pro^. With the goal of relieving high-energy inter-atom contacts stemming from the initialization of four current systems, 3000-cycle steepest descent minimization followed by 3000-cycle conjugate gradient minimization were carried out to optimize each system. Next, a 200-ps heating process from 0 to 310 K was implemented on each system in the canonical ensemble (NVT), with a weak harmonic restriction of 2 kcal·mol^−1^·Å^2^ exerted on heavy atoms and subsequently the system was equilibrated for another 200 ps at the temperature of 310 K. Then, a 200-ps density equilibrium process was conducted under the isothermal-isobaric ensemble (NPT) and the temperature of 310 K so that the density of systems was kept at 1.01 g/cm^3^, in which the Berendsen barostat was applied to maintain constant pressure (P = 1 atm) [92]. After that, three independent 2-ns conventional MD simulations were performed at the NVT to deeply relax the system. Three ending structures from conventional MD simulations were wielded to run an equilibrium of 5 ns to obtain the starting structures for performing three independent GaMD simulations of 1 μs on each system in the NPT ensemble (T = 310 K, P = 1 atm) without restraints, of which the initial atomic velocities of each structure were assigned through the Maxwell distribution. GaMD simulations can efficiently enhance conformation sampling of targets through smoothing the potential energy surface of the system by increasing the harmonic enhancement potential and the details for GaMD simulations have been clarified in the Miao’s work [43] and Supporting Information File S1. Coordinates were recorded every 2000 steps and 750,000 frames were saved for post-processing analysis. The program PyReweighting1.0 from Miao et al. was wielded to reweight the original free energy of the four current 3CL^pro^-related systems [93]. The chemical bonds linking hydrogen atoms with heavy atoms were restrained with the SHAKE algorithm [94]. The temperatures of the four 3CL^pro^-related systems were regulated using a Langevin thermostat [95] with a collision frequency of 1.0 ps^−1^. The nonbonded interactions between atoms were calculated through the periodic boundary conditions and the particle mesh Ewald method (PME) with a 12 Å cutoff [96]. In our work, all simulations were triggered utilizing the program pmemd.cuda [97,98] in Amber 22. Three independent GaMD trajectories were integrated by a single trajectory for facilitating post-processing analysis using the CPPTRAJ programs [99]. The PCA and calculations of dynamics cross-correlation maps (DCCMs) were conducted by means of the Bio3D package [64,65,66] and the details for PCA have been explained in our previous work [48].

### 3.3. Free Energy Landscapes

To investigate inhibitor-mediated impacts on free energy profiles of 3CLpro, the projections (PC1 and PC2) of GaMD trajectories onto the first two eigenvectors were used as reaction coordinates (RCs) to construct FELs. In GaMD simulations, the free energy $\left(A\right)=-k_{B}Tln\left(\rho_{A}\right)$ of systems is reweighted as

(1) $$F\left(A\right)=F^{*}\left(A\right)-\sum_{k=1}^{2}\frac{\beta^{k}}{k!}C_{k}+F_{C}$$

in which $F^{*}\left(A\right)=-k_{B}Tlnp^{*}\left(A\right)$ is the modified free energy stemming from GaMD simulations, $F_{C}$ indicates a constant and $k_{B}T$. The probability distribution $p^{*}\left(A\right)$ of selected RCs from GaMD simulations can be reweighted to recover the canonical ensemble distribution $\rho_{A}$. All calculations in the free energy reweighting were realized by using the program PyReweighting developed by Miao et al. and the detail for the reweighting procedure has been clarified in the work of Miao et al. [93].

### 3.4. Calculation of MM-GBSA and QM/MM-GBSA

Molecular mechanics Poisson−Boltzmann surface area (MM-PBSA) and MM-GBSA are thought to be two efficient approaches to quickly compute the binding free energies of inhibitors or drugs to targets [67,68,69]. In light of the evaluation of the performance of these two methods [100,101,102,103], the performance of the MM-GBSA and MM-PBSA methods shows difference in different biomacromolecule systems. Thus, we computed binding free energies of inhibitor-3CL^pro^ according to the following Equation (2) to simply compare their results.

(2) $$\Delta G_{bind}=\Delta H-T\Delta S$$

where ∆*H* and −*T*∆*S* represent the binding enthalpy and entropy, respectively. The entropy changes (−*T*∆*S*) were estimated by using the mmpbsa_py_nabnmode program [104] in Amber 22. In this work, ∆*H* is calculated by using the MM-GBSA and QM/MM-GBSA methods. In the calculations of MM-GBSA, ∆*H* is further divided into four separate components indicated in Equation (3):

(3) $$\Delta H=\Delta E_{vdW}+\Delta E_{ele}+\Delta G_{egb}/\Delta G_{epb}+\Delta G_{esurf}$$

in which $\Delta E_{vdW}$ and $\Delta E_{ele}$ are van der Waals and electrostatic interactions, which are calculated using molecular mechanics, respectively. $\Delta G_{egb}/\Delta G_{epb}$ and $\Delta G_{esurf}$ indicate polar solvation free energy and nonpolar solvation free energy. In QM/MM-GBSA calculations, ∆H can be expressed in Equation (4)

(4) $$\Delta H=\Delta E_{vdW}+\Delta E_{ele}+\Delta G_{egb}/\Delta G_{epb}+\Delta G_{esurf}+\Delta G_{escf}$$

in this calculation, inhibitors and residue (C145) involved in hydrogen bonding interactions (HBIs) were described at the QM region, in which $\Delta G_{escf}$ is treated using the semi-empirical Hamiltonian PM6 method. For calculations of MM-GBSA and QM/MM-GBSA, $\Delta G_{egb}$ is calculated by using the generalized Born (GB) model [105] while $\Delta G_{esurf}$ is estimated with the empirical equation $\Delta G_{esurf}=\gamma \times \Delta \mathrm{SASA}+\beta$, from which γ and β are set as 0.0072 kcal·mol^−1^·Å^−2^ and 0.0 kcal·mol^−1^ in the calculations of MM-GBSA and QM/MM-GBSA calculations, respectively, while they are set as 0.00542 kcal·mol^−1^·Å^−2^ and 0.92 kcal·mol^−1^ in the MM-PBSA calculations [106]. In this current work, we used 200 snapshots to perform calculations of MM-PBSA, MM-GBSA and QM/MM-GBSA.

### 3.5. Normal Mode Analysis and Correlation Network Analysis

The NMA of 3CL^pro^ was realized with the Bio3d package. The normal modes of the separate structure were produced through solving the eigenvalue equation

(5) $$V^{T}KV=\lambda$$

in which K indicates the effective force-constant Hessian matrix. The principal modes (normal modes) of movements were characterized by the eigenvector $V_{k}$ and their corresponding eigenvalues $\lambda_{k}$. The energetic costs corresponding to the displacement of 3CL^pro^ along the eigenvectors were described by the eigenvalues. Fluctuations from the NMA of each whole 3CL^pro^ were estimated to reflect amplitudes of the absolute atomic motion. The square-fluctuations and cross-correlations of fluctuations of modes were analyzed by means of the Bio3D package.

Correlation network analysis (CNA) is an efficient tool for recognizing protein regions with correlated motions. For this analysis, a weighted graph is built, in which each residue represents a node and the weight of the connection between nodes *i* and *j* reflects their corresponding cross-correlation value $C_{ij}$ expressed by either the Pearson-like form [62] or the linear mutual information [107]. The correlation matrix is computed by the equation $C_{ij}=<\Delta r_{i}\Delta \Delta r_{j}>/{\left(<\Delta r_{i}^{2}><\Delta r_{j}^{2}>\right)}^{1/2}$, in which $\Delta r_{i}$ and $\Delta r_{j}$ represents the displacement of the *i*th and *j*th Cα atoms away from their averaged positions. Then, edges are mapped onto residue pairs with $C_{ij}\geq C_{0}$ across all ensemble structures. After that, edge weights are computed by using $-\log\left(<C_{ij}>\right)$, of which 〈⋅〉 indicates the ensemble average [108]. Then, Girvan and Newman betweenness clustering [109] was carried out to yield aggregate nodal clusters or communities being highly intra-connected but loosely inter-connected. Finally, visualization of the resulting network and community structures in 3D models were displayed by utilizing the program VMD1.9.3 [110].

### 3.6. Molecular Docking

The crystal structure of APO 3CL^pro^ taken from 7JVZ (https://www.rcsb.org/structure/7JVZ (12 January 2024)) was used as a starting structure to dock 7YY, 7XB and Y6G into 3CL^pro^. The docked structures were minimized to relieve the high-energy contacts between the atoms. For our work, the grid box in the (x, y, z) direction was assigned as (60, 60, 60) Å with a spacing value of 0.375 Å. The Lamarckian genetic algorithm (LGA) used in the software AutoDock Vina 1.2.6 [111] and the default parameters of the software were adopted to perform our current docking by using the first twenty binding free energies extracted (Table S1) to compare with the previous MM-GBSA and QM/MM-GBSA calculations. The docked structures of inhibitor-3CL^pro^ complexes with the first twenty high scores were displayed in Figure S9, which display different binding poses. The molecular docking was performed using high-throughput molecular dynamics (HTMD1.2.1) [112] and AutoDock Vina1.2.6 [111].

## 4. Conclusions

Insights into the molecular mechanisms underlying conformation responses between structural domains and inhibitor-3CL^pro^ binding can provide useful information for drug design targeting 3CL^pro^. Three independent GaMD simulations, each running for 1 μs, have been performed on four current systems to improve conformation samplings of 3CL^pro^. Our simulations show that the presence of inhibitors evidently changes structural flexibility and internal dynamics of 3CL^pro^ and contacting extents of 3CL^pro^ with solvents. The results from the CNA indicate that the conformational responses of catalytic regions between domains I and II caused by inhibitor bindings can be transferred into domain III through a node in domain II, which affects the activity of 3CL^pro^. FELs constructed by using the principal components PC1 and PC2 from the PCA as RCs suggest that inhibitor binding changes free energy files of 3CL^pro^. Furthermore, the results from the NMA and PCA show that the binding of inhibitors alters structural fluctuations and mobility of catalytic regions and domain III, which may exert influences on the activity of 3CL^pro^. MM-GBSA and QM/MM-GBSA calculations indicate that 7YY has the strongest inhibiting ability among our selected inhibitors, thus its molecular scaffold can be used for further optimizing molecular structure to design new efficient 3CL^pro^ inhibitors. Meanwhile, van der Waals interactions are the main forces in inhibitor-3CL^pro^ binding, which is worth noting in future drug design toward 3CL^pro^. Hot interaction spots L27, H41, C44, S46, M49, N142, G143, S144, C145, H163, H164, M165 and E166, identified by residue-based free energy decomposition, can be used as efficient targets of anti-COVID-19 drug design and aid development of clinically available 3CL^pro^ inhibitors.

## Figures and Tables

**Table 1:** Binding free energies of inhibitors to 3CP^pro^ calculated using MM-GBSA method.

^a^ Components	7YY-3CL^pro^		7XB-3CL^pro^		Y6G-3CL^pro^	
	Average	Std	Average	Std	Average	Std
$\Delta E_{vdW}$	−53.29	0.31	−47.67	0.43	−51.21	0.16
$\Delta E_{ele}$	−37.05	0.38	−34.43	0.51	−23.48	0.30
$\Delta G_{egb}$	54.39	0.29	50.08	0.44	40.12	0.35
$\Delta G_{esurf}$	−5.76	0.02	−6.51	0.04	−5.72	0.02
^b^ ΔH	−41.71	0.27	−41.52	0.45	−40.29	0.26
−TΔS	27.05	0.74	27.63	0.71	26.02	1.00
^c^ $\Delta G_{bind}$	−14.66	0.33	−13.89	0.41	−14.27	0.54
^d^ $\Delta G_{exp}$	−10.78		−6.92		−9.34	

^a^ All free energy components are scaled in kcal/mol; ^b^ $\Delta H=\Delta E_{vdW}+\Delta E_{ele}+\Delta G_{egb}+\Delta G_{esurf}$; ^c^ $\Delta G_{bind}=\Delta H-T\Delta S$; ^d^ The experimental values were transformed from the experimental IC50 values in references [25,59] with the equation $\Delta G_{exp}=-RTlnIC50$.

**Table 2:** Binding free energies of inhibitors to 3CP^pro^ calculated using QM/MM-GBSA.

^a^ Components	7YY-3CL^pro^		7XB-3CL^pro^		Y6G-3CL^pro^	
	Average	Std	Average	Std	Average	Std
$\Delta E_{vdW}$	−49.28	0.24	−47.13	0.43	−50.89	0.23
$\Delta E_{ele}$	−0.1	0.01	−0.05	0.01	−0.06	0.01
$\Delta G_{egb}$	59.39	0.29	49.92	0.48	51.55	0.26
$\Delta G_{esurf}$	−5.75	0.02	−5.69	0.04	−5.78	0.04
$\Delta G_{escf}$	−44.75	0.32	−35.56	0.55	−32.67	0.34
^b^ ΔH	−40.5	0.22	−39.31	0.55	−37.85	0.21
−TΔS	27.05	1.00	27.63	0.71	26.02	1.00
^c^ $\Delta G_{bind}$	−13.45	0.21	−11.68	0.30	−11.83	0.22
^d^ $\Delta G_{exp}$	−10.78		−6.92		−9.34	

^a^ All free energy components are scaled in kcal/mol; ^b^ $\Delta H=\Delta E_{vdW}+\Delta E_{ele}+\Delta G_{egb}+\Delta G_{esurf}+\Delta G_{escf}$; ^c^ $\Delta G_{bind}=\Delta H-T\Delta S$; ^d^ The experimental values were transformed from the experimental IC50 values in references [25,59] with the equation $\Delta G_{exp}=-RTlnIC50$.

## Data Availability

Data are contained within the article and Supplementary Materials.

## Supplementary Materials

The following supporting information can be downloaded at https://www.mdpi.com/article/10.3390/molecules30040805/s1. File S1. The details for GaMD simulations; Table S1. Binding free energies of inhibitors to 3CP^pro^ calculated using MM-PBSA method; Table S2. Binding free energies of inhibitors to 3CL^pro^ derived from molecular docking (kcal/mol); Table S3. Hydrogen bonds between inhibitors and 3CL^pro^ analyzed using the CPPTRAJ module; Table S4. Free energy decomposition of key residues calculated using MM-GBSA method; Figure S1. The function of RMSDs as simulation times; Figure S2. Difference in RMSFs between inhibitor-bound 3CL^pro^ and the APO one; Figure S3. The evolution of solvent accessible surface area of four systems as the simulation time; Figure S4. Information alterations of 7YY-bound 3CL^pro^ captured by principal component analysis; Figure S5. Information alterations of 7XB-bound 3CL^pro^ detected by principal component analysis; Figure S6. Information alterations of Y6G-bound 3CL^pro^ captured by principal component analysis; Figure S7. Concerted motions of structural regions near catalytic sites; Figure S8. Free energy profile and representative structures of APO 3CL^pro^; Figure S9. Dynamics cross-correlation maps of 3CL^pro^ calculated by using the Cα atoms; Figure S10. Network communications between key structural domains; Figure S11. Structures of inhibitor-3CL^pro^ docking with different binding poses.
