# Supplementary material for: Identifying Inhibitor-SARS-CoV2-3CLpro Binding Mechanism Through Molecular Docking, GaMD Simulations, Correlation Network Analysis and MM-GBSA Calculations
Source: Molecules. 2025 Feb 10;30(4):805. doi: 10.3390/molecules30040805 (PMC11857935; doi:10.3390/molecules30040805)
Supplement: Supplementary file 1 [file molecules-30-00805-s001.zip › molecules-3409675-supplementary.pdf]

Identifying Inhibitor-SARS-CoV2-3CL^{pro} Binding Mechanism Through Molecular Docking, GaMD Simulations, Correlation Network Analysis and MM-GBSA Calculations

Jianzhong Chen*, Jian Wang, Wanchun Yang, Lu Zhao and Xiaoyan Xu

School of Science, Shandong Jiaotong University, Jinan 250357, China; jzchen@sdjtu.edu.cn or chenjianzhong1970@163.com (J.C.); wangjian_lxy@sdjtu.edu.cn (J.W.); yangwch1982@126.com (W.Y.); zhaolusdu@163.com (L.Z.); xuxiaoyan@sdjt.edu.cn (X.X.)
*Correspondence: jzchen@sdjtu.edu.cn or chenjianzhong1970@163.com (J.C.)

File S1. The details for GaMD simulations

In GaMD simulations, if the potential energy $V(\vec{r})$ of the system is lower than a threshold energy E, the $V(\vec{r})$ is upgraded into $V^*(\vec{r})$ according to two equations (S1) and (S2)

$$V^*(\vec{r}) = V(\vec{r}) + \Delta V(\vec{r}) \quad \text{(S1)}$$

$$\Delta V(\vec{r}) = \begin{cases} 0, & V(\vec{r}) \geq E \\ \frac{1}{2}k\left(E - V(\vec{r})\right)^2, & V(\vec{r}) < E \end{cases} \quad \text{(S2)}$$

where the parameter k represents the harmonic force constant. Through two enhanced sampling principles described in the equations (S3) and (S4), the parameters E and k can be tuned

$$V_{max} \leq E \leq V_{min} + \frac{1}{k} \quad \text{(S3)}$$

$$\mathrm{k} = k_0 \frac{1}{V_{max} - V_{min}} \quad \text{(S4)}$$

in which if E is set as the lower bound $E = V_{max}$, then k_0 is obtained by using the equation (S5)

$$k_0 = \min\left(1.0, \frac{\sigma_0}{\sigma_V} \cdot \frac{V_{max} - V_{min}}{V_{max} - V_{avg}}\right) \quad \text{(S5)}$$

on the contrary, if E is set as the upper bound $E = V_{min} + \frac{1}{k}$, then k_0 is got according to the equation (S6)

$$k_0 = \left(1.0 - \frac{\sigma_0}{\sigma_V}\right) \cdot \left(\frac{V_{max} - V_{min}}{V_{avg} - V_{min}}\right) \quad \text{(S6)}$$

in the aforementioned equations, three energy parameters V_{max}, V_{min} and V_{avg} respectively indicate the maximum, minimum and averaged potential energies of the systems extracted from three independent cMD simulations. The parameter σ_V corresponds to the standard deviation of the system potential energies and the σ_0 is a user-determined upper limit for rationally reweighting.

Table S1. Binding free energies of inhibitors to $3CP^{pro}$ calculated using MM-PBSA method

[a]Components	7YY-$3CL^{pro}$		7XB-$3CL^{pro}$		Y6G-$3CL^{pro}$	
	Average	std	Average	std	Average	std
ΔE_{vdW}	-53.29	0.31	-47.67	0.43	-51.21	0.16
ΔE_{ele}	-37.05	0.38	-34.43	0.51	-23.48	0.30
ΔG_{epb}	60.40	0.30	57.21	0.48	47.01	0.45
ΔG_{surf}	-3.16	0.02	-3.91	0.04	-3.12	0.02
[b]ΔH	-33.1	0.34	-28.8	0.49	-30.8	0.31
$-T\Delta S$	27.05	0.74	27.63	0.71	26.02	1.00
[c]ΔG_{bind}	-6.05	0.36	-1.17	0.45	-4.78	0.57
[d]ΔG_{exp}	-10.78		-6.92		-9.34	

[a]All free energy components are scaled in kcal/mol; [b]$\Delta H = \Delta E_{vdW} + \Delta E_{ele} + \Delta G_{epb} + \Delta G_{esurf}$; [c]$\Delta G_{bind} = \Delta H - T\Delta S$; [d]The experimental values were transformed from the experimental IC50 values in references [1,2] with the equation $\Delta G_{exp} = -RTlnIC50$

Table S2. Binding free energies of inhibitors to $3CL^{pro}$ derived from molecular docking (kcal/mol).

Modes	7YY-$3CL^{pro}$	7XB-$3CL^{pro}$	Y6G-$3CL^{pro}$
1	-10.2	-8.8	-9.2
2	-9.6	-8.2	-8.9
3	-9.4	-7.9	-8.5
4	-9.3	-7.7	-8.4
5	-9.1	-7.6	-8.3
6	-9.1	-7.6	-8.2
7	-8.7	-7.6	-8.1
8	-8.7	-7.6	-8.1
9	-8.4	-7.5	-8.0
10	-8.4	-7.5	-7.8
11	-8.4	-7.5	-7.8
12	-8.3	-7.4	-7.6
13	-8.3	-7.4	-7.6
14	-8.3	-7.2	-7.6
15	-8.2	-7.1	-7.5
16	-8.0	-7.0	-7.5
17	-7.9	-7.0	-7.5
18	-7.9	-7.0	-7.5
19	-7.8	-7.0	-7.4
20	-7.7	-6.8	-7.3

Table S3. Hydrogen bonds between inhibitors and $3CL^{pro}$ analyzed using the CPPTRAJ module

Complexes	Hydrogen bonds	^{a}Distance(Å)	^{a}Angle(°)	^{b}Occupacy(%)
	7YY-O09…C145-N-H	3.04	151.10	87.48
	7YY-O04…H163-NE2-HE2	3.12	138.66	73.68
7YY-$3CL^{pro}$	7YY-N10…G143-N-H	3.21	156.21	60.29
	7YY-O09…G143-N-H	2.97	134.19	57.36
	7YY-O09…S144-N-H	2.96	134.08	46.64
	7XB-O08…C145-N-H	3.10	149.04	42.31
	7XB-O04…H163-NE2-HE2	2.94	141.85	39.29
7XB-$3CL^{pro}$	7XB-O08…G143-N-H	2.92	141.06	46.77
	7XB-O09…G143-N-H	3.18	158.77	16.32
	7XB-O35…E166-N-H	3.02	155.89	77.65
	Y6G-O01…E166-N-H	3.04	160.86	79.97
	Y6G-N11…G143-N-H	3.29	135.69	17.34
Y6G-$3CL^{pro}$	Y6G-N12…G143-N-H	3.31	134.94	17.34
	Y6G-N11…S146-H-H	3.22	137.88	18.54
	Y6G-N11…C145-N-H	3.29	136.44	11.69

^{a}Hydrogen bonds are analyzed by an acceptor···donor distance of < 3.5 Å and acceptor···H-donor angle of > 120°; ^{b}Occupancy (%) is defined as the percentage of simulation time that a specific hydrogen bond exists.

Table S4. Free energy decomposition of key residues calculated using MM-GBSA method[a].

Complexes	Residue	ΔS_{vdW}	ΔB_{vdW}	ΔT_{vdW}	ΔS_{ele}	ΔB_{ele}	ΔT_{ele}	ΔS_{gb}	ΔB_{gb}	ΔT_{gb}	[b]ΔG
	L27	-1.06	-0.07	-1.13	-0.05	-0.26	-0.31	0.1	0.29	0.39	-1.17
	H41	-1.55	-0.33	-1.87	-0.78	-0.40	-1.18	0.98	0.57	1.55	-1.67
	M49	-1.42	-0.08	-1.50	0.02	0.07	0/09	0.2	-0.03	0.17	-1.49
	N142	-1.95	-0.42	-2.88	-0.49	-1.71	-2.2	1.64	0.88	2.51	-3.0
7YY-$3CL^{pro}$	G143	-0.32	-0.47	-0.79	0.25	-3.24	-2.99	-0.21	1.96	1.75	-2.12
	S144	-0.76	-0.24	-1.0	-0.03	-1.87	-1.89	0.15	0.75	0.9	-2.02
	C145	-1.27	-0.26	-1.53	-0.3	-1.28	-1.58	0.45	0.46	0.91	-2.32
	H163	-0.04	-0.78	-0.83	-2.58	-0.01	-2.59	1.55	-0.0	1.55	-1.9
	M165	-1.04	-0.63	-1.66	-0.16	-0.67	-0.83	0.25	0.51	0.76	-1.87
	H41	-1.74	-0.27	-2.01	-0.15	-1.11	-1.26	0.93	0.86	1.79	-1.71
	M49	-1.8	-0.13	-1.93	-0.13	0.13	-0.0	0.45	-0.11	0.34	-1.88
	N142	-1.75	-0.62	-2.37	-2.19	-0.93	-3.12	2.74	0.42	3.17	-2.64
	G143	-0.19	-0.21	-0.4	0.19	-2.79	-2.6	-0.16	1.57	1.41	-1.65
7XB-$3CL^{pro}$	S144	-0.6	-0.3	-0.9	0.04	-1.07	-1.03	0.01	0.51	0.53	-1.43
	C145	-1.04	-0.3	-1.34	-0.05	-0.81	-0.86	0.21	0.22	0.42	-1.92
	H163	-0.56	-0.07	-0.63	-2.94	-0.11	-3.05	1.83	0.15	1.98	-1.74
	M165	-1.69	-0.61	-2.29	-0.04	-0.81	-0.84	0.21	0.17	0.39	-2.96
	E166	-1.27	-0.4	-1.67	-1.1	-1.03	-2.13	2.23	0.67	2.9	-1.14
	H41	-1.42	-0.34	-1.77	-1.02	-1.36	-2.38	1.62	1.31	2.94	-1.35
	C44	-0.38	-0.07	-0.45	0.16	-2.61	-2.45	-0.08	1.74	1.67	-1.26
	S46	-0.57	-0.45	-1.02	-0.23	-0.21	-0.44	0.44	0.19	0.63	-1.0
Y6G-$3CL^{pro}$	M49	-2.15	-0.17	-2.32	-0.5	0.05	-0.45	0.63	-0.11	-0.52	-2.5
	N142	-1.34	-0.55	-1.89	0.03	-0.43	-0.4	0.87	0.42	1.28	-1.32
	M165	-1.76	-0.69	-2.45	-0.4	-2.04	-2.44	0.48	0.61	1.09	-4.01
	E166	-1.75	-0.79	-2.54	0.35	0.65	1.0	0.93	-0.27	0.66	-1.24

[a]All free energy components are scaled in kcal/mol; [b]ΔS_{vdW} and ΔB_{vdW} are the energy contributions of sidechain and backbone to van der Waals interactions, respectively. ΔS_{ele} and ΔB_{ele} separately correspond to the energy contributions of sidechain and backbone to electrostatic interactions; ΔS_{gb} and ΔB_{gb} represent the energy contributions to polar solvation free energies of complexes, individually.

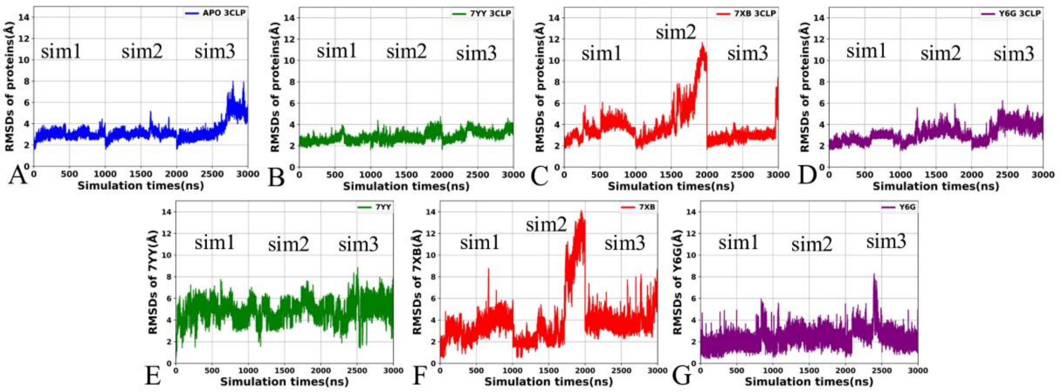

Figure S1. The function of RMSDs as simulation times: (A) RMSDs of backbone atoms from APO 3CL^{pro}, (B) RMSDs of backbone atoms from 7YY-bound 3CL^{pro}, (C) RMSDs of backbone atoms from 7XB-bound 3CL^{pro}, (D) RMSDs of backbone atoms from Y6G-bound 3CL^{pro}, (E) RMSDs of all heavy atoms from 7YY, (F) RMSDs of all heavy atoms from 7XB and (G) RMSDs of all heavy atoms from Y6G. In this figure, sim1, sim2 and sim3 represent three independent GaMD simulations of 1 μs.

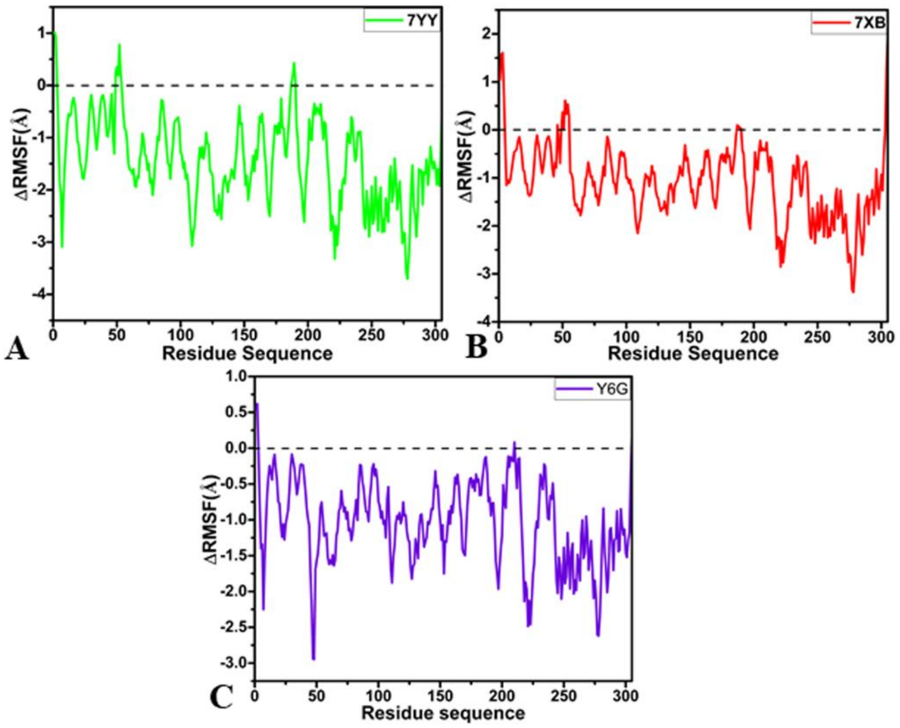

Figure S2. Difference in RMSFs between inhibitor-bound 3CL^{pro} and the APO one: (A) 7YY, (B) 7XB and (C) Y6G.

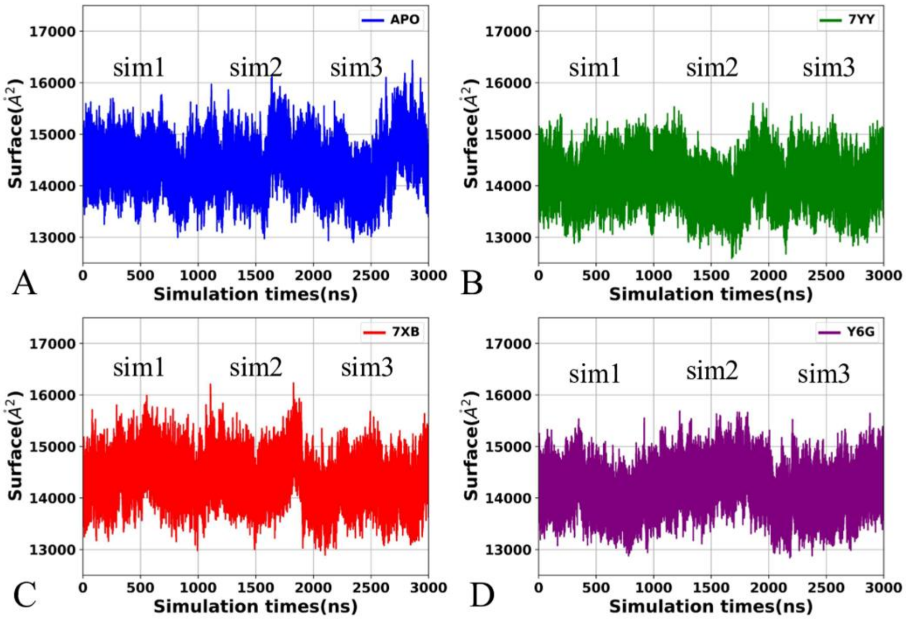

Figure S3. The evolution of solvent accessible surface area of four systems as the simulation time: (A) APO 3CL^{pro}, (B) 7YY-bound 3CL^{pro}, (C) 7XB-bound 3CL^{pro} and (D) Y6G-bound 3CL^{pro}.

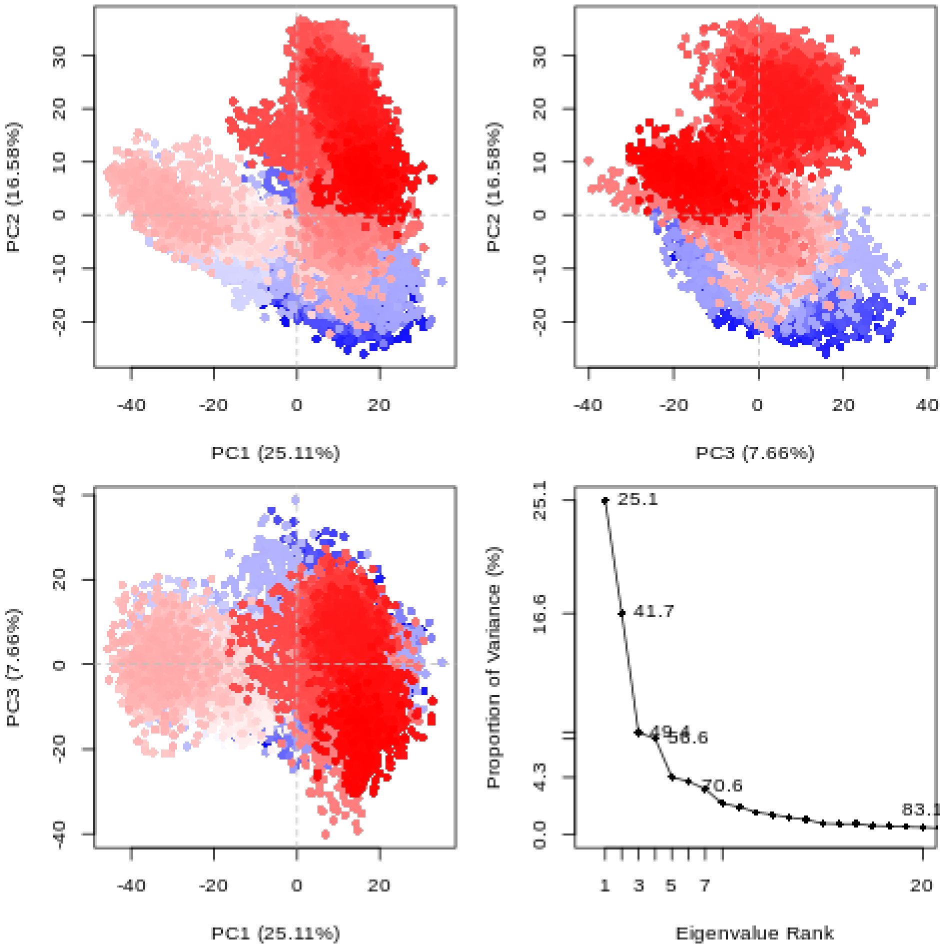

Figure S4. Information alterations of 7YY-bound 3CL^{pro} captured by principal component analysis. The first three principal components PC1-PC3 account for fluctuating regions with 49.4% of overall fluctuations. In this figure, the blue region describes the most significant movements, while the red region reflects the least flexible motions. The transformation from the blue to the red implies conformation transition and the pale red or pale blue regions embody intermediate states during conformation transition.

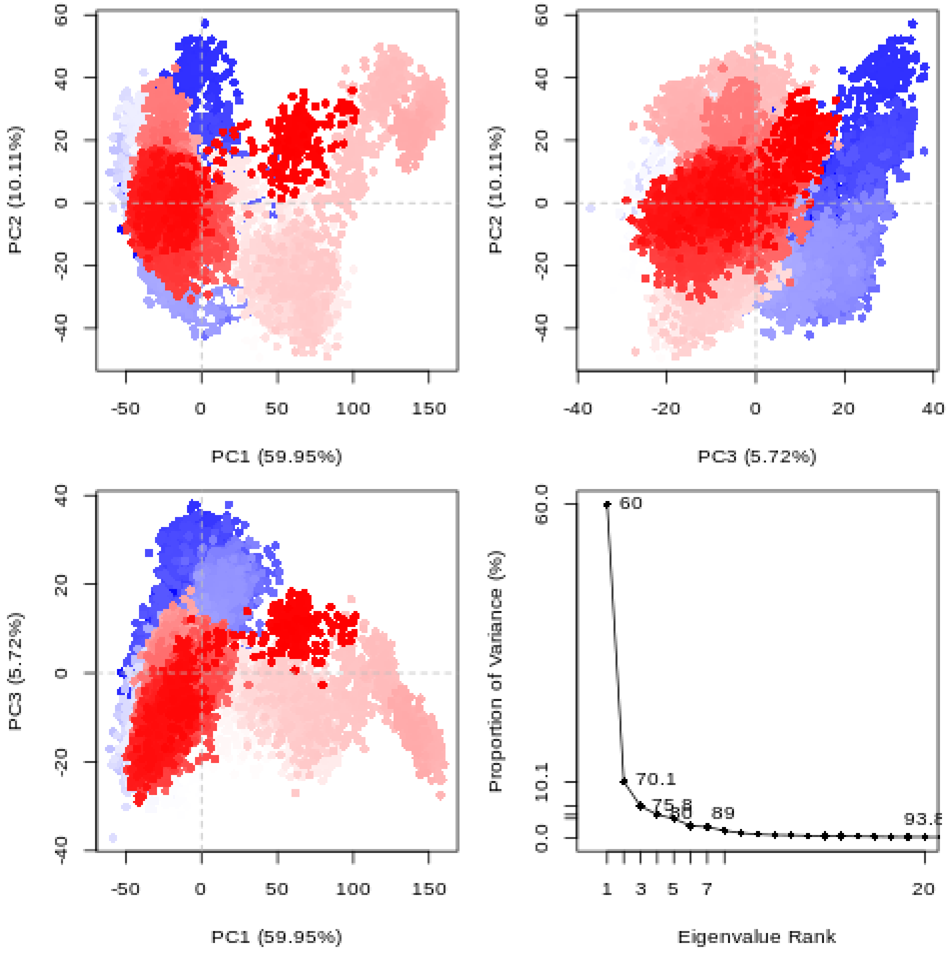

Figure S5. Information alterations of 7XB-bound 3CL^{pro} detected by principal component analysis. The first three principal components PC1-PC3 account for fluctuating regions with 75.8% of overall fluctuations. In this figure, the blue region describes the most significant movements, while the red region reflects the least flexible motions. The transformation from the blue to the red implies conformation transition and the pale red or pale blue regions embody intermediate states during conformation transition.

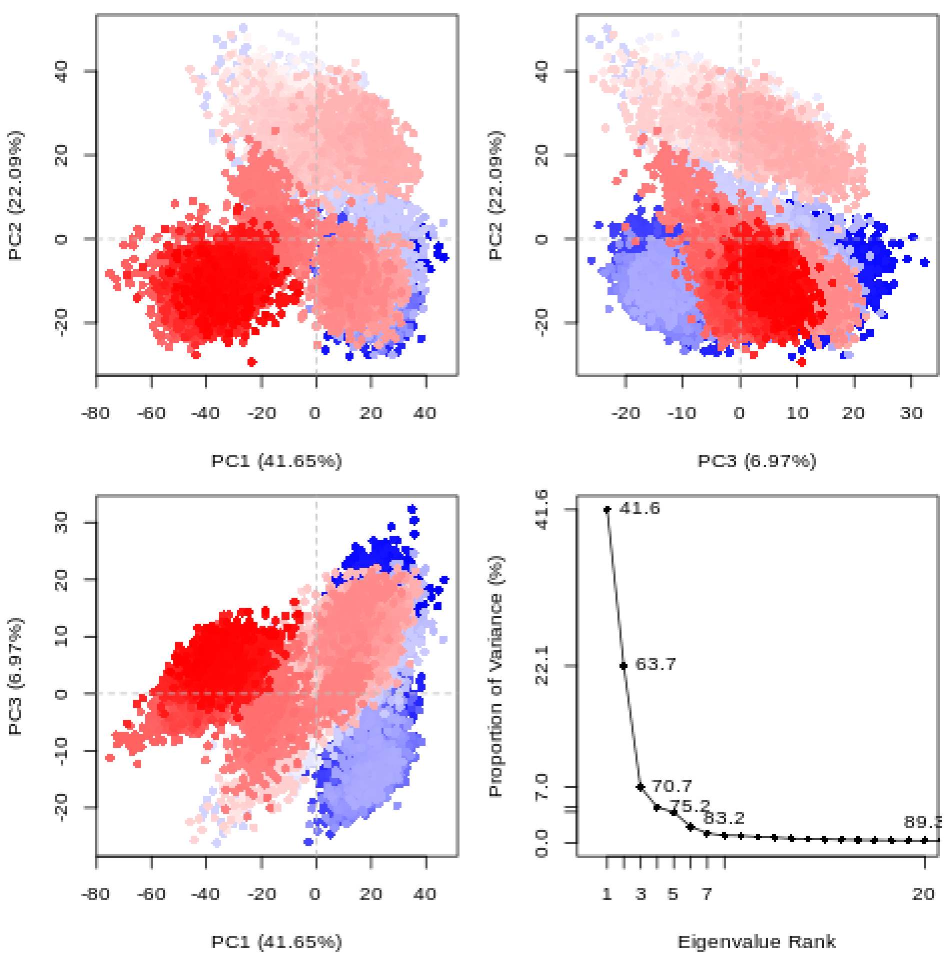

Figure S6. Information alterations of Y6G-bound 3CL^{pro} captured by principal component analysis. The first three principal components PC1-PC3 account for fluctuating regions with 75.2% of overall fluctuations. In this figure, the blue region describes the most significant movements, while the red region reflects the least flexible motions. The transformation from the blue to the red implies conformation transition and the pale red or pale blue regions embody intermediate states during conformation transition.

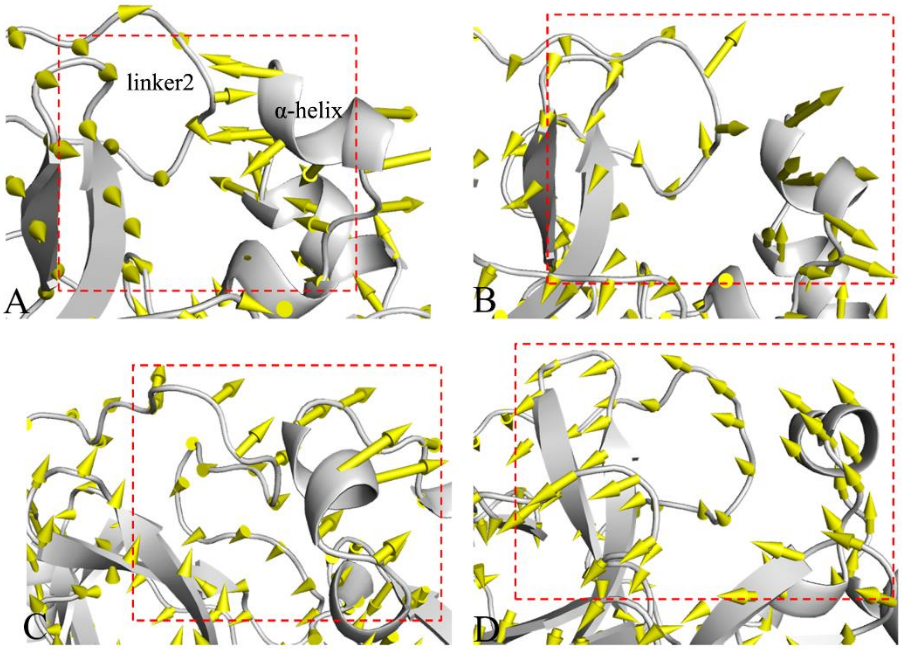

Figure S7. Concerted motions of structural regions near catalytic sites: (A) APO 3CL^{pro}, (B) 7YY-bound 3CL^{pro}, (C) 7XB-bound 3CL^{pro} and (D) Y6G-bound 3CL^{pro}.

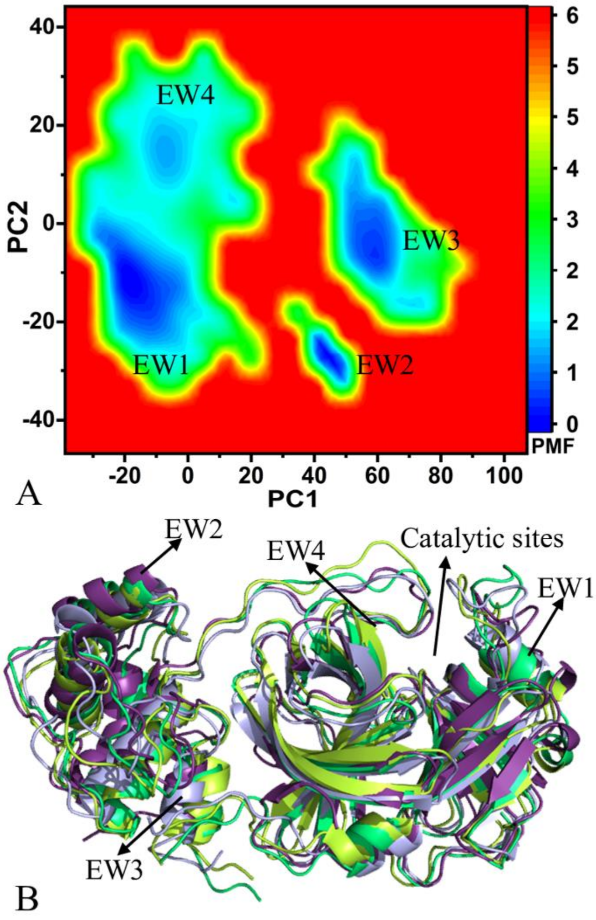

Figure S8. Free energy profile and representative structures of *APO* 3CL^{pro}: (A) free energy landscape and (B) alignments of four representative structures EW1-EW4. The PMF is scaled in kcal/mol.

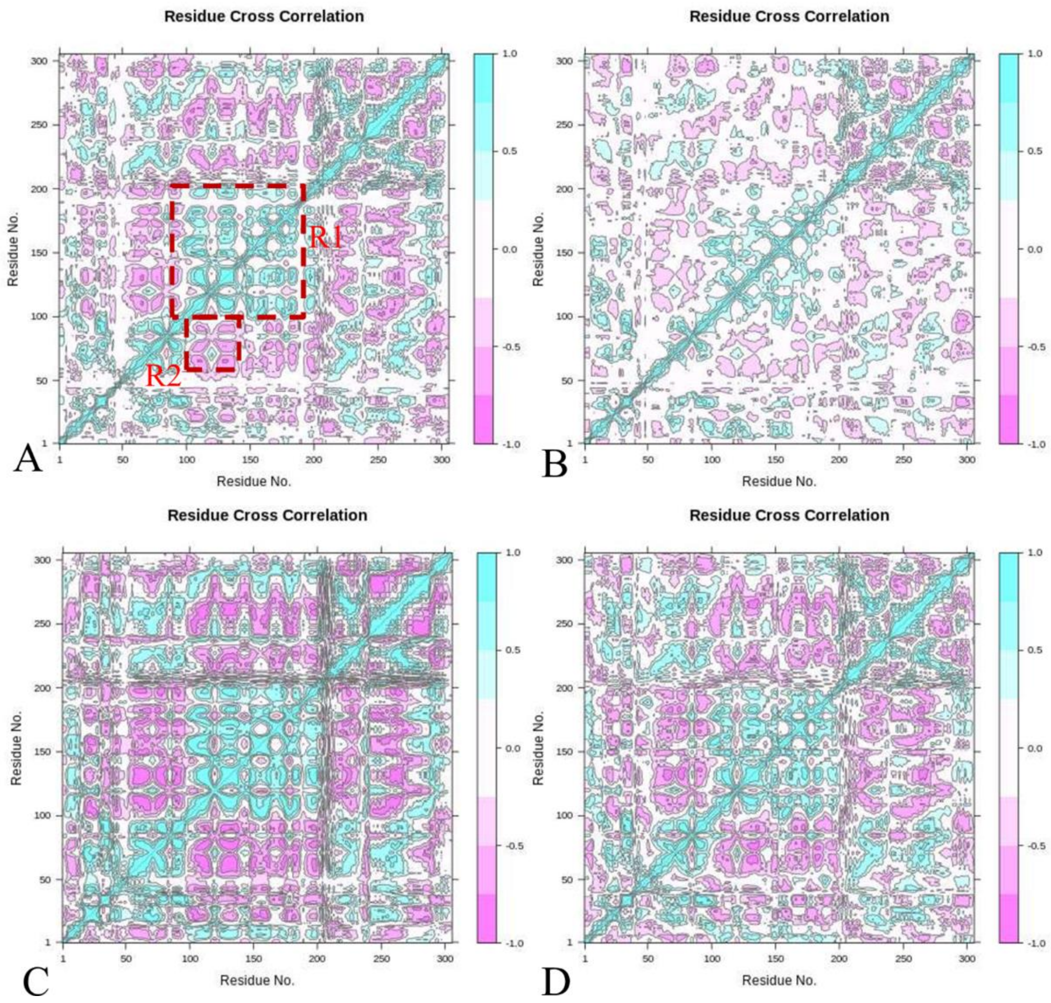

Figure S9. Dynamics cross-correlation maps of $3CL^{pro}$ calculated by using the Cα atoms: (A) *APO* $3CL^{pro}$, (B) 7YY-bound $3CL^{pro}$, (C) 7XB-bound $3CL^{pro}$ and (D) Y6G-bound $3CL^{pro}$. In this figure the violet and cyan respectively indicate the most anti-correlated and positive correlated motions.

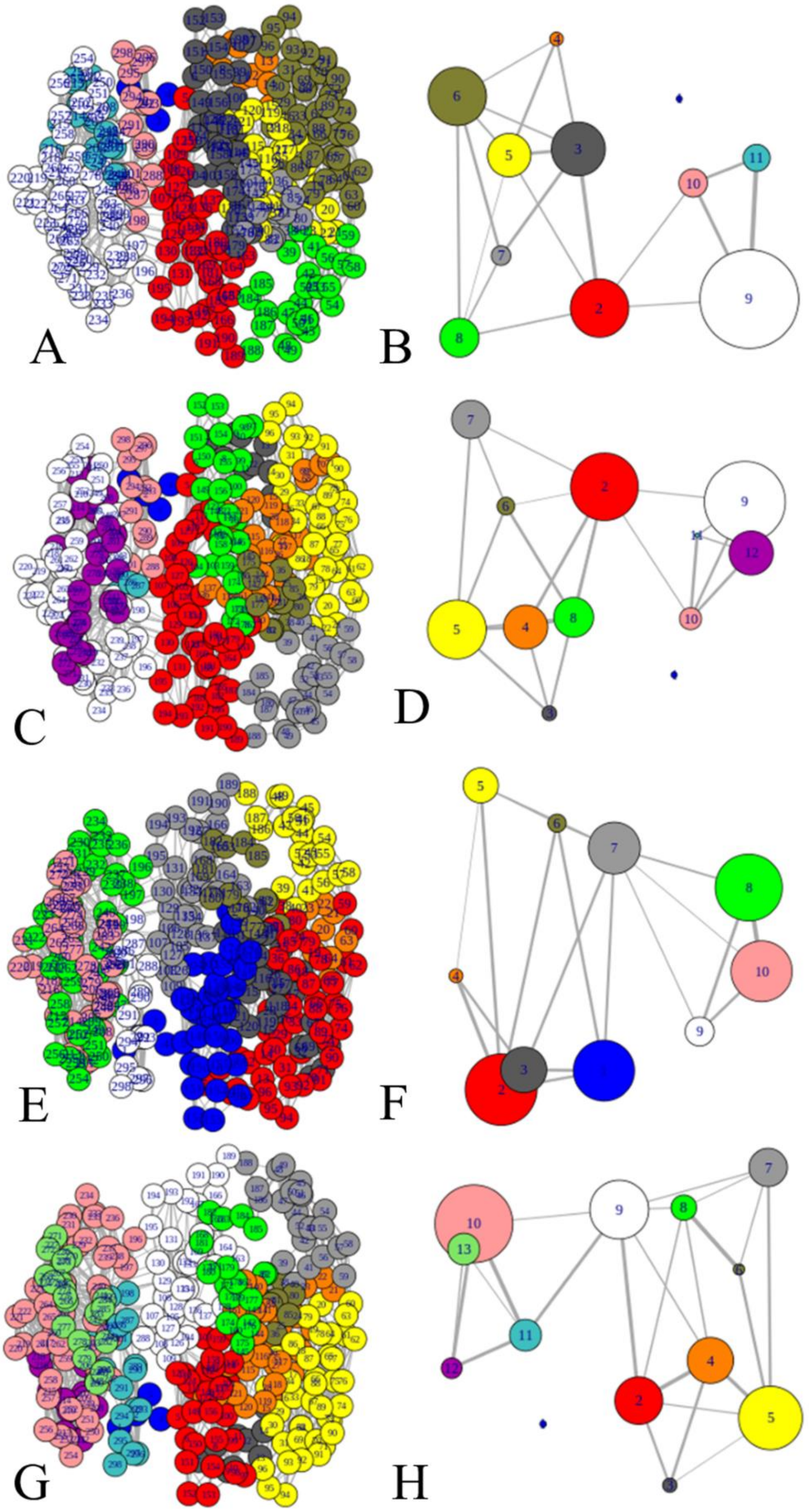

Figure S10. Network communications between key structural domains: (A) residue network of *APO* 3CL^{pro}, (B) cluster network of *APO* 3CL^{pro}, (C) residue network of 7YY-bound 3CL^{pro}, (D) cluster network of 7YY-bound 3CL^{pro}, (E) residue network of 7XB-bound 3CL^{pro}, (F) cluster network of 7XB-bound 3CL^{pro}, (G) residue network of Y6G-bound 3CL^{pro}, (D) cluster network of Y6G-bound 3CL^{pro}.

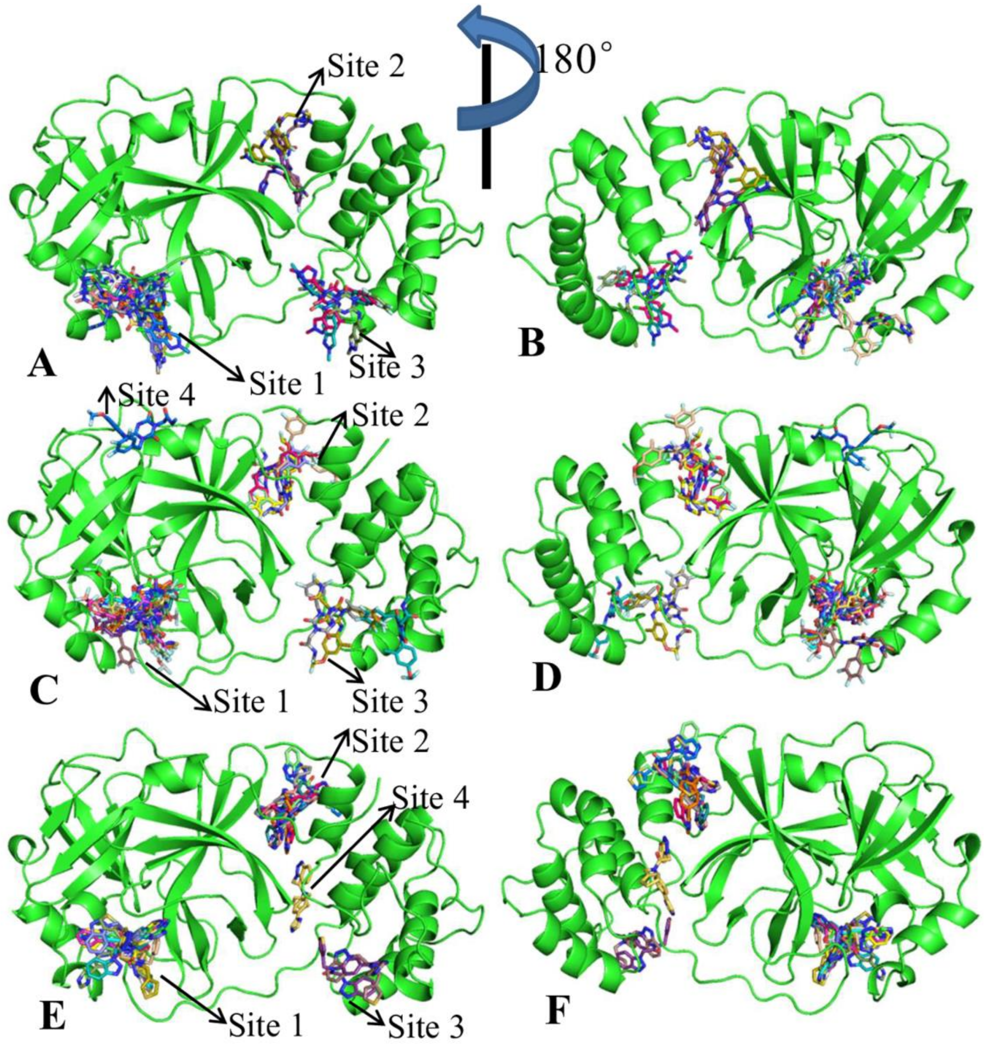

Figure S11. Structures of inhibitor-3CL^{pro} docking with different binding poses: (A) and (B) 7YY-3CL^{pro} docking and rotation of 180° between them; (C) and (D) 7XB-3CL^{pro} docking and rotation of 180° between them and (E) and (F) Y6G-3CL^{pro} docking and rotation of 180° between them.

Reference

1. Unoh, Y.; Uehara, S.; Nakahara, K.; Nobori, H.; Yamatsu, Y.; Yamamoto, S.; Maruyama, Y.; Taoda, Y.; Kasamatsu, K.; Suto, T. et al. Discovery of S-217622, a Noncovalent Oral SARS-CoV-2 3CL Protease Inhibitor Clinical Candidate for Treating COVID-19. J. Med. Chem. 2022, 65, 6499-6512.
2. Han, S. H.; Goins, C. M.; Arya, T.; Shin, W.-J.; Maw, J.; Hooper, A.; Sonawane, D. P.; Porter, M. R.; Bannister, B. E.; Crouch, R. D. et al. Structure-Based Optimization of ML300-Derived, Noncovalent Inhibitors Targeting the Severe Acute Respiratory Syndrome Coronavirus 3CL Protease (SARS-CoV-2 3CLpro). J. Med. Chem. 2022, 65, 2880-2904.
